# Unraveling the Self-Assembly of the *Pseudomonas aeruginosa* XcpQ Secretin Periplasmic Domain Provides New Molecular Insights into Type II Secretion System Secreton Architecture and Dynamics

**DOI:** 10.1128/mBio.01185-17

**Published:** 2017-10-17

**Authors:** Badreddine Douzi, Nhung T. T. Trinh, Sandra Michel-Souzy, Aline Desmyter, Geneviève Ball, Pascale Barbier, Artemis Kosta, Eric Durand, Katrina T. Forest, Christian Cambillau, Alain Roussel, Romé Voulhoux

**Affiliations:** a Aix Marseille University, CNRS, IMM, LISM, Marseille, France; b Aix Marseille University, CNRS, AFMB, Marseille, France; c Aix Marseille University, INSERM, CRO2, Marseille, France; d Aix Marseille University, CNRS, IMM, Marseille, France; e University of Wisconsin—Madison, Madison, Wisconsin, USA; Max Planck Institute for Terrestrial Microbiology

**Keywords:** *Pseudomonas aeruginosa*, secretin, dynamics, protein structure-function, stoichiometry, type II secretion system

## Abstract

The type II secretion system (T2SS) releases large folded exoproteins across the envelope of many Gram-negative pathogens. This secretion process therefore requires specific gating, interacting, and dynamics properties mainly operated by a bipartite outer membrane channel called secretin. We have a good understanding of the structure-function relationship of the pore-forming C-terminal domain of secretins. In contrast, the high flexibility of their periplasmic N-terminal domain has been an obstacle in obtaining the detailed structural information required to uncover its molecular function. In *Pseudomonas aeruginosa*, the Xcp T2SS plays an important role in bacterial virulence by its capacity to deliver a large panel of toxins and degradative enzymes into the surrounding environment. Here, we revealed that the N-terminal domain of XcpQ secretin spontaneously self-assembled into a hexamer of dimers independently of its C-terminal domain. Furthermore, and by using multidisciplinary approaches, we elucidate the structural organization of the XcpQ N domain and demonstrate that secretin flexibility at interdimer interfaces is mandatory for its function.

## IMPORTANCE

Bacterial secretins are large homooligomeric proteins constituting the outer membrane pore-forming element of several envelope-embedded nanomachines essential in bacterial survival and pathogenicity. They comprise a well-defined membrane-embedded C-terminal domain and a modular periplasmic N-terminal domain involved in substrate recruitment and connection with inner membrane components. We are studying the XcpQ secretin of the T2SS present in the pathogenic bacterium *Pseudomonas aeruginosa*. Our data highlight the ability of the XcpQ N-terminal domain to spontaneously oligomerize into a hexamer of dimers. Further *in vivo* experiments revealed that this domain adopts different conformations essential for the T2SS secretion process. These findings provide new insights into the functional understanding of bacterial T2SS secretins.

## INTRODUCTION

Bacterial secretins constitute the outer membrane (OM) component of several distinct transenvelope nanomachines often found in pathogenic bacteria and dedicated to the secretion, extrusion, and/or assembly of large extracellular macromolecules (1). For example, secretins allow the secretion of large folded virulence factors by the type II secretion system (T2SS) or the emergence on the cell surface of large pilus and needle appendages assembled by the type 4 pilus assembly system (T4PS) and the type III secretion system (T3SS), respectively. Secretins are large homooligomeric assemblies of 12 to 16 monomers forming a distinctive bipartite ring-shaped and cylindrical structure on their C- and N-terminal sides, respectively (1).

The C-terminal region, or β-domain, is highly conserved among all secretins and formed by a giant 56- to 64-β-stranded pore constituted by the 14 to 16 4-stranded β-sheets of each subunit (2–4). This domain forms the OM portal through which secreted substrates transit to the cell surface and is believed to be the major determinant in oligomer formation and stability (5, 6).

The periplasmic N domain of secretins is constituted by the succession of structurally independent subdomains labeled N0 to N3 (7–9). In contrast to the C domain, their number and structural organization are variable and dependent on the nanomachine. While four N0, N1, N2, and N3 subdomains are found in T2SS secretins, T4PS and T3SS secretins are shorter due to the absence of N1-N2 and N2 subdomains, respectively. N1 to N3 share structural similarities with each other and are characterized by an α-β fold interconnected by short loops (7–10). High-resolution three-dimensional (3D) structures of N domains are available for at least 4 secretins, 2 from T2SS (7, 8), 1 from T3SS (9), and 1 from T4P (11). Structural comparisons between secretin N domains show that despite low sequence identity, the individual subdomains are structurally very similar. In all cases, the N domain of secretins is thought to protrude deep into the periplasm, where it interacts with the inner membrane (IM) part of the related nanomachine. T2SS N domains have indeed been shown to directly bind the IM protein GspC (12–14), thus forming a continuous tunnel crossing the whole periplasm. A similar transperiplasmic structure has been isolated for the T3SS and T4PS nanomachines (15, 16). In addition, secretin N domains also play a direct role in secretion processes, since direct interactions have been reported with the secreted substrates (12, 17, 18) or the assembled pilus (17, 19).

So far, only full-length or C-terminal domains of bacterial secretins have been found to form oligomers, suggesting that secretins acquire their quaternary structure through their C-terminal domains. In contrast, the precise oligomeric arrangement of secretin N domains has never been experimentally demonstrated and was so far predicted only from full-length electron microcopy (EM) 3D structures. In the last three reported cases, each presenting a nearly atomic-resolution structure of a secretin C domain, similar symmetry based on the monomer asymmetric unit was proposed for the N domain (C1 symmetry) (2–4). An alternate arrangement based on the oligomerization of dimers of secretin subunits (C2 symmetry) has, however, also been reported for at least three other secretins (8, 20, 21).

Here, we revealed that the N-terminal domain of the T2SS secretin XcpQ from *Pseudomonas aeruginosa* is able to self-assemble into a hexamer of dimers independently of its C-terminal domain. Based on its structural organization, we used nanobody interference and cysteine cross-linking to determine the N domain stoichiometric, dynamics, and interactomic significance in the global *in vivo* context of the T2SS nanomachine.

## RESULTS

### *In vitro* self-oligomerization of purified XcpQ_N012_.

**FIG 1:**
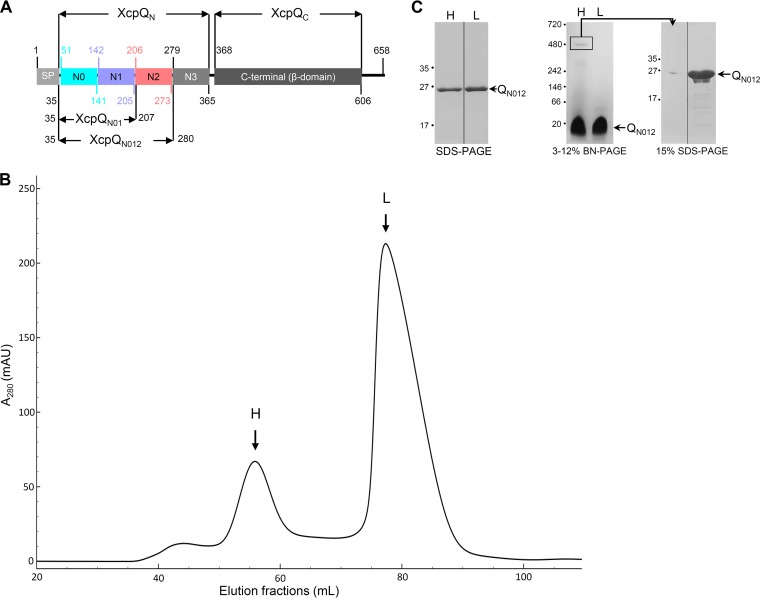
XcpQ_N012_ forms homomultimers in solution. (A) Schematic representation of XcpQ secretin subdomains and their boundaries (amino acid number on the pre-XcpQ protein). The signal peptide (SP) and the XcpQ_N012_ and XcpQ_N01_ variants are also represented (3, 8). (B) Size exclusion chromatography (SEC) of the purified XcpQ_N012_. The elution volume (from a HiLoad 16/600 Superdex 200 column) is plotted on the *x* axis, and the 280-nm absorbance is plotted on the *y* axis. mAU, milli-absorbance units. (C) Coomassie blue-stained SDS-PAGE and 3 to 12% Tris-glycine blue-native PAGE (BN-PAGE) analysis of the high (H)- and low (L)-molecular weight complexes. The excised band from BN-PAGE corresponding to the identified H complex was analyzed by 15% SDS-PAGE. The electrophoretic profile shows that XcpQ_N012_ is the only protein forming the H complex compared to the purified XcpQ_N012_ loaded in the neighboring lane. For SDS-PAGE and BN-PAGE, molecular mass markers (in kilodaltons) are indicated on the left.

In *P. aeruginosa*, the C-terminal domain of the secretin XcpQ (XcpQ_C_) encompasses residues 368 to 606, whereas the N-terminal domain (XcpQ_N_) covers residues 51 to 365 (Fig. 1A) (3, 8). We previously showed that purified XcpQ_N_ interacts with secreted effectors and the inner membrane partner XcpP in a 1:1 stoichiometry (12). As a next step toward its structural determination, we purified to homogeneity a large amount of XcpQ_N012_ domain and analyzed it by size exclusion chromatography (SEC) (see Text S1 in the supplemental material). We observed that the protein is eluted in two well-defined high-molecular-weight (H) and a low-molecular-weight (L) complexes exclusively constituted by the XcpQ_N012_ protein (Fig. 1B and C). Further analysis of H and L fractions under native conditions confirms that XcpQ_N012_ forms a high-molecular-weight complex whose excision and analysis under denaturing conditions validate the homomultimeric constitution (Fig. 1C). The calibration of the SEC column estimates the apparent molecular masses of the H and L species at about 310 and 55 kDa, respectively (Fig. S1). Since XcpQ_N012_ has a molecular mass of 26 kDa, we estimate that H and L complexes are homododecamers (12-mer) and homodimers (2-mer), respectively. To confirm these oligomeric states of XcpQ_N012_, we performed sedimentation velocity experiments (see Text S1 and Fig. S2A and B). The *C*(*S*) distribution of the sedimentation coefficient revealed the presence of two well-defined species that sediment at 2.1S ± 0.4S, corresponding to 61% of the loading concentration, and at 7.0S ± 0.6S, corresponding to 15% of the loading concentration (Fig. S2A). Between these two well-defined peaks, we observed a small shoulder centered at approximately 3.7S corresponding to an intermediate oligomeric species. The molecular masses estimated from this sedimentation velocity are 56 ± 7 kDa and 329 ± 15 kDa for the two majority species (Fig. S2B) and are compatible with dimers and dodecamers, thus confirming the two species isolated during SEC purification.

10.1128/mBio.01185-17.1 TEXT S1  Additional information on materials and methods used in this study. Download TEXT S1, DOCX file, 0.02 MB. Copyright © 2017 Douzi et al. 2017 Douzi et al. This content is distributed under the terms of the Creative Commons Attribution 4.0 International license.

10.1128/mBio.01185-17.3 FIG S1  Analysis of the oligomeric state of XcpQ_N012_ by SEC. (A) SEC curves using calibration standard proteins. The curve of the XcpQ_N012_ was superimposed with calibration curves. The elution volume (from a HiLoad 16/600 Superdex 200 column) is plotted on the *x* axis, and the 280-nm absorbance is plotted on the *y* axis. The initials for each calibration standard protein are mentioned as well as the corresponding molecular weight. (B) HiLoad 16/600 Superdex 200 calibration. The log (MW) of standard proteins is plotted on the *x* axis, and the calculated *V*_*e*_/*V*_*o*_ is plotted on the *y* axis. Each dark triangle indicates the position of each protein used for the calibration. The red triangles indicate the positions of H and L species. Download FIG S1, PDF file, 0.2 MB. Copyright © 2017 Douzi et al. 2017 Douzi et al. This content is distributed under the terms of the Creative Commons Attribution 4.0 International license.

10.1128/mBio.01185-17.4 FIG S2  **A**nalysis of the oligomeric state of XcpQ_N012_ by AUC and EM. (A) Coefficient sedimentation distribution *C*(*S*) of 7 mg/ml of XcpQ_N012_ obtained by sedimentation velocity experiments. (B) The *C*(*M*) distribution function obtained from these similar experiments indicates the presence of species with molecular masses compatible with a dimer (L) and a dodecamer (H). (C) Negative-stain EM images of XcpQ_N012_ oligomeric complex purified by SEC. The insets show two class averages generated by EMAN2 from 166 picked particles. Scale bar (20 nm) is shown in white. Download FIG S2, PDF file, 0.2 MB. Copyright © 2017 Douzi et al. 2017 Douzi et al. This content is distributed under the terms of the Creative Commons Attribution 4.0 International license.

### Structural organization of XcpQ_N012_ homodimer.

**FIG 2:**
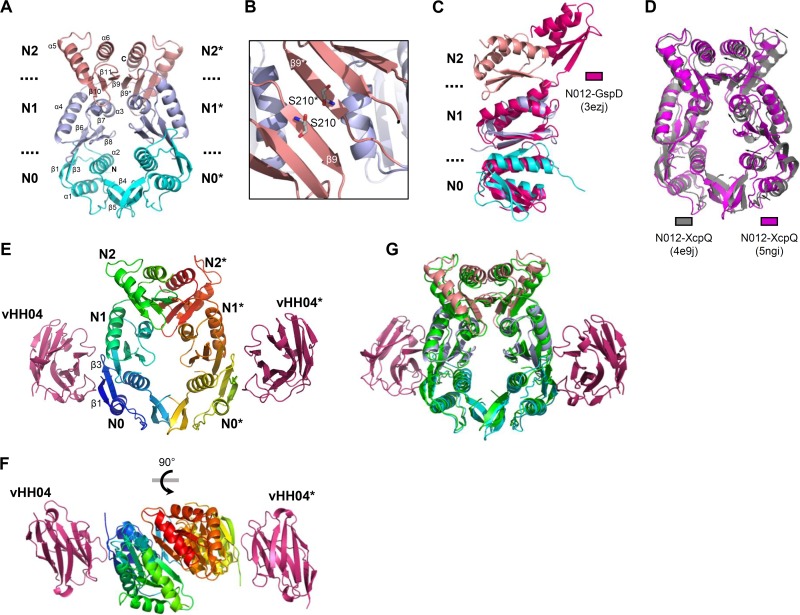
High-resolution 3D structures of XcpQ_N012_ and vHH04-XcpQ_N012_ determined by X-ray crystallography. (A) Ribbon view of the XcpQ_N012_ homodimer. The N0, N1, and N2 subdomains of each monomer are colored in cyan, light blue, and pink, respectively. (B) Zooming in on a top view of the dimer interface highlights the face-to-face assembly driven by the N2-N2* interface. The S210 residues selected for cysteine point substitution at the N2-N2* interface are shown in stick form. (C) Cartoon representation of XcpQ_N012_ with the N-terminal domain of GspD (PDB ID 3EZJ) superimposed. (D) Cartoon representation in side view of the XcpQ_N012_ structures: PDB ID 5NGI (purple) (present study) versus PDB ID 4E9J (gray) (8). The higher packing of the present dimer is indicated by arrows. (E and F) Side view (E) and top view (F) of the vHH04-XcpQ_N012_ complex structure. The vHH04 nanobodies are colored in hot pink. (G) Superimposition of the XcpQ_N012_ structure in free state (see panel A legend for color code) or in complex with vHH04 nanobody (green).

To gain deeper structural information on XcpQ_N012_ multimerization, we endeavored to determine the 3D structure of the purified recombinant XcpQ_N012_ by X-ray crystallography. The overall structure of the XpQ_N012_ monomer, refined at 3.0-Å resolution, showed a typical organization into three subdomains, N0, N1, and N2, where each subdomain abuts the next in an N0:N1:N2 arrangement in three dimensions (Fig. 2A and Table 1). The crystal structure revealed an axial dimeric association of XcpQ_N012_ organized in face-to-face fashion (Fig. 2A). The dimer packing is stabilized by the interaction between N0-N0* and N2-N2* subdomains from each monomer (the “*” corresponds to the second molecule in the dimer). The dimerization seems to be driven by the N2-N2* contact with a buried surface of 600 Å^2^ that represents about 60% of the total buried surface. Most of the contacts between N2 subdomains are localized within the β9 strand interacting with the β9* in the facing N2* subdomain, creating a continuous 6-stranded antiparallel β-sheet (Fig. 2B and Table 2). Two other structures of N-terminal domains of T2SS secretin have been solved for *Escherichia coli* GspD and *P. aeruginosa* XcpQ secretins (7, 8). In contrast to XcpQ_N012_, the N2 subdomain of GspD is not in contact with the N1 subdomain. This difference results in a high root mean square deviation (RMSD) (4.36 Å) when the two structures are superimposed (Fig. 2C). The structural comparison of XcpQ_N012_ with the same three subdomains resolved previously in a different space group (Protein Data Bank [PDB] identifier [ID] 4E9J) (8) shows that the two structures adopt similar folds with an RMSD value of 1.9 Å (Fig. 2D). However, a slight difference was observed between the packing of the two XcpQ dimers, due to a general displacement of monomer B toward monomer A (Fig. 2D). This leads, in the present study, to a more compact dimer with a larger buried surface area of 1,040 Å^2^ compared to 880 Å^2^ for the previous structure. However, it cannot be excluded that this compactness results from overall crystal packing effects.

**TABLE 1:** Data collection and refinement statistics of XcpQ_N012_ and XcpQ_N012_-vHH04 complex^a^

Data collection and refinement parameters	XcpQ_N012_	XcpQ_N012_-vHH04
Data collection		
PDB ID	5NGI	5MP2
Source	Soleil PX 1	Soleil PX 2
Space group	P2_1_	P1
Cell (Å; °)	*a* = 40.4, *b* = 122.25, *c* = 55.44, β = 109.0	*a* = 40.06, *b* = 63.79, *c* = 76.05, α = 104.37, β=100.61, γ = 108.04
No. of monomers	2	2/2
Resolution limits (Å)	48.2–2.98 (3.16–2.98)	38.75–2.9 (3.13–2.9)
*R*_merge_	0.058 (0.70)	0.134 (0.53)
CC1/2	0.998 (0.896)	0.989 (0.45)
Unique reflections	10,400 (1,659)	14,042 (2,926)
Mean (*I*) (SD)	14.9 (2.0)	6.2 (1.4)
Completeness (%)	99.3 (98.2)	95.2 (97.2)
Multiplicity	4.5 (4.4)	2.0 (2.1)
		
Refinement		
Resolution (Å)	48.2–2.98 (3.33–2.98)	38.75–2.9 (3.13–2.9)
No. of reflections	10,394 (2,791)	14,039 (2,923)
No. of protein/water/ion atoms	3,022/13	4,904/124/0
No. of test set reflections	520	732
*R*_work_/*R*_free_	0.203/0.228 (0.249/0.315)	0.204/0.253 (0.246/0.38)
RMSD bonds (Å)/angles (°)	0.010/1.23	0.012/1.20
B-Wilson/B-mean Å	125/137	71/61.9
Ramachandran: preferred/allowed/outliers (%)	96.4/3.6/0	96.8/3.2/0

^a^ Numbers in parentheses refer to the highest-resolution bin.

**TABLE 2:** Residues involved in hydrogen bonds in XcpQ_N012_ homodimer and vHH04-XcpQ_N012_ association

Interface	First component		Second component	
	Residue no.	Atom	Residue no.	Atom
N0-N0*	R82	NH1	A117	O
	V115	O	N127	ND2
	Q118	OE1	Q118	NE2
	R123	NH1	Q118	OE1
	N127	NH2	V115	O
				
N1-N2*	E158	OE1	N213	ND2
				
N2-N2*	Y209	O	V211	H
	Y209	OH	R251	NH1
	S210	OG	S210	OG
	V211	O	Y209	H
	N213	ND2	D208	OD1
	R251	NH1	Y209	OH
				
vHH04:N0	Y33	OH	N56	HD2
	Y59	HH	S91	O
	I104	O	T54	H

### XcpQ_N012_ homododecamers form ring-shaped structures.

**FIG 3:**
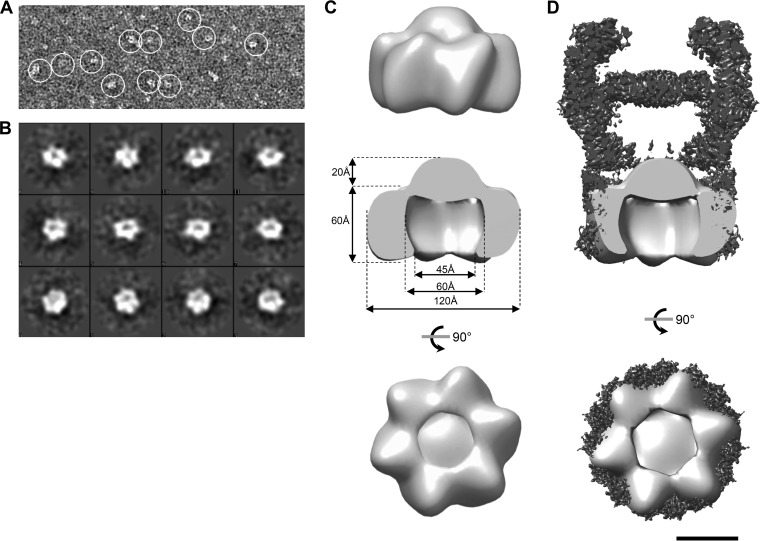
Low-resolution EM model of XcpQ_N012_ dodecamers. (A) Representative micrograph of the data set used for image processing. White circles indicate isolated Trx-XcpQ_N012_ dodecamers. (B) Gallery of representative class averages generated by EMAN2 after 2D classification. (C) Top, side, and bottom views of the three-dimensional reconstruction model of the XcpQ_N012_ dodecamer obtained by electron microscopy (accession number EMD-3641). (D) Side and bottom cutout views of the cryo-EM structure of GspD from *E. coli* K-12 (EMD-6675) colored in black with the low-resolution structure of XcpQ_N012_ colored in light gray superimposed. Bar, 5 nm.

Our SEC data revealed that the XcpQ_N012_ domain is able to self-assemble into dodecamers. We therefore assessed the structural organization of this dodecameric complex by negative-staining transmission electron microscopy (TEM). Most of the two-dimensional (2D) class averages generated from individual particles were ring shaped but not homogenous enough to build a model (Fig. S2C). To circumvent the instability of matured XcpQ_N012_, we analyzed by TEM the purified fusion protein, Trx-XcpQ_N012_, also able to form a dodecameric complex in solution (Fig. S3). TEM analysis of the Trx-XcpQ_N012_ SEC fractions revealed numerous ring-shaped particles (Fig. 3A and B). In order to structurally characterize the preformed ring complex composed by Trx-XcpQ_N012_, we generated a 3D model of the complex at a resolution of 30 Å (Fig. 3C). The 3D reconstitution revealed a right-handed ring-shaped structure with an external diameter of 120 Å and a height of 80 Å, including a protruding conical top of 20 Å. A cutout view of the resulting model revealed an internal cavity with a 60-Å maximum diameter that constricts to 45 Å at the base (Fig. 3C). Interestingly, if we consider the dodecameric stoichiometry of the complex, each asymmetric unit should be constituted by the homodimer. Those dimensions are, moreover, in agreement with the external diameter of the periplasmic part of the membrane-extracted full-length secretin GspD (EMD-6675) (Fig. 3D) (3), thus strengthening the physiological relevance of these dodecameric structures.

10.1128/mBio.01185-17.5 FIG S3  Trx-XcpQ_N012_ assembles into a dodecameric complex. SEC of the purified Trx-XcpQ_N012_. The elution volume (from a Superdex 200 16/600 column) is plotted on the *x* axis, and the 280-nm absorbance is plotted on the *y* axis. Left upper inset is the Coomassie blue-stained SDS-PAGE analysis of the H and L fractions. (Right) Estimation of the MW of the H and L species of Trx-Q_N012_ using Superdex 200 16/600 calibration. Dark triangles indicate the position of each protein used for the calibration. The red triangles indicate the positions of H and L species. The estimated MW is indicated on the SEC profile. Download FIG S3, PDF file, 0.2 MB. Copyright © 2017 Douzi et al. 2017 Douzi et al. This content is distributed under the terms of the Creative Commons Attribution 4.0 International license.

### XcpQ_N012_ assembles into a hexamer of dimers.

The ability of XcpQ_N012_ to self-assemble into both dimers and dodecamers suggests that six dimers constitute the building blocks of the dodecameric complex. Based on previous work (8), we engineered a point cysteine substitution within the N2-N2* interface at position S210, involved in the dimeric interface by establishing a hydrogen bond with the S210 residue from the facing monomer (Fig. 2B). The XcpQN_012_-S210C variant was purified to homogeneity by SEC in the presence of the oxidative reagent H_2_O_2_, which triggers disulfide bridge formation. Under such oxidative conditions, the SDS-PAGE analysis of the “H” SEC fractions of XcpQN_012_-S210C clearly showed that this variant forms dodecamers constituted by cross-linked dimers via the S210C disulfide bridge (Fig. S4A). The direct involvement of the N2 subdomain in XcpQ_N012_ oligomeric assembly was confirmed by the monomeric recovery of the XcpQ_N01_ variant, even at high concentrations (Fig. S5). Our results demonstrate that XcpQ_N012_ dodecamerization is a sequential process driven by an N2-mediated dimerization followed by the gathering of six dimers into a hexamer of dimers.

10.1128/mBio.01185-17.6 FIG S4  XcpQN_012_-S210C and XcpQ_N012_-T54C-Q86C form oligomers in the dodecameric complex under oxidative conditions. SEC of the purified XcpQN_**012**_-S210C (A) and XcpQ_**N012**_-T54C-Q86C (B) variants. The fractions corresponding to the dodecameric complex of each variant indicated by arrows were analyzed by Coomassie blue-stained SDS-PAGE under reducing and nonreducing conditions (+ and – β-mercaptoethanol [β-ME]). XcpQN_012_-S210C and XcpQ_N012_-T54C-Q86C dimers and higher oligomers are indicated by arrows. The nonspecific band recovered above 95 kDa in SEC experiments is indicated by a star (*). Molecular mass markers (in kDa) are indicated on the left. Download FIG S4, PDF file, 0.2 MB. Copyright © 2017 Douzi et al. 2017 Douzi et al. This content is distributed under the terms of the Creative Commons Attribution 4.0 International license.

10.1128/mBio.01185-17.7 FIG S5  The N2 subdomain is essential for XcpQ_N012_ dodecamer assembly. SEC profile of XcpQ_N012_ (black line) was superimposed on XcpQ_N01_ (red line). The SEC profile of the protein standards is also shown in dashed lines. The calibration curve as well the position of the estimated MW of XcpQ_N01_ (red triangle) is shown in the right panel. Download FIG S5, PDF file, 0.2 MB. Copyright © 2017 Douzi et al. 2017 Douzi et al. This content is distributed under the terms of the Creative Commons Attribution 4.0 International license.

Like Trx-XcpQ_N012_, Trx-XcpQ_N012_-S210C also forms ring-shaped structures as shown by TEM (Fig. S6A and B). 3D reconstitution allows us to obtain a model at 25-Å resolution. The overall structure presents a height of 120 Å and a width of 120 Å (Fig. S6C). A cutout view showed an internal cavity with a 55-Å diameter. The structure can be divided into two parts: the upper part resembles the model obtained for Trx-XcpQ_N012_, while the lower part, with a height of 30 Å, probably corresponds to Trx, which was not visible in the model generated for Trx-XcpQ_N012_ (Fig. S6C). The ability to identify the volume corresponding to Trx with the S210C variant could be explained by the low flexibility of the N2 domain due to the disulfide bridge formation. Such a position of Trx validates the orientation of N and C termini in the EM structure. Moreover, the similarity between the Trx-XcpQ_N012_ and Trx-XcpQ_N012_-S210C structures confirms that the N-terminal domain of XcpQ is formed by the hexamerization of the dimeric building block revealed by the crystal structure.

10.1128/mBio.01185-17.8 FIG S6  Low-resolution EM model of XcpQN_012_-S210C. (A) Representative micrograph of the data set used for image processing. White circles indicate isolated Trx-XcpQ_N012_-S210C dodecamers. (B) Gallery of representative class averages generated by EMAN2 after 2D classification. (C) Top, side, and bottom views of the three-dimensional reconstruction model of the XcpQN_012_-S210C dodecamer obtained by electron microscopy (accession no. EMD-3649). The three-dimensional reconstitution of XcpQ_N012_ colored in violet is also shown. Scale bar (5 nm) is shown in black. Download FIG S6, PDF file, 0.4 MB. Copyright © 2017 Douzi et al. 2017 Douzi et al. This content is distributed under the terms of the Creative Commons Attribution 4.0 International license.

### Identification of XcpQ_N012_ interdimer interface by nanobody costructure determination and interference experiments.

As part of a project to generate camel antibodies against Xcp T2SS components, we identified a specific nanobody (vHH04) directed against XcpQ_N_. This nanobody was cloned, produced, and purified according to a procedure previously described (22) and briefly presented in the supplemental material (see Text S1). Affinity chromatography experiments revealed that the histidine-tagged vHH04 is able to copurify with untagged XcpQ_N012_ using magnetic nickel beads (Fig. S7A). Further affinity measurement using biolayer interferometry (BLi) revealed a dissociation constant (*K*_*D*_) of 5.6 µM between vHH04 and XcpQ_N012_ (Fig. S7B). Moreover, when added prior to the concentration step that triggers XcpQ_N012_ oligomerization, vHH04 permits dimerization but prevents hexamerization (Fig. S7C). These competition experiments suggest that the vHH04 binding interface overlaps the XcpQ_N012_ interface involved in dodecamer formation. Interestingly, when vHH04 is mixed with the preformed XcpQ_N012_ multimer, the SEC profile revealed that vHH04 is able to bind XcpQ_N012_ dodecamers. This is shown by the slight shift to the left of the corresponding peak fractions in the presence of vHH04 (Fig. S7D). The estimated mass of the XcpQ_N012_-vHH04 complex was around 100 kDa higher than the mass of the XcpQ_N012_ dodecamer, suggesting that only six vHH04s are associated with the XcpQ_N012_ complex (6 by ~16 kDa). We are therefore proposing that only one XcpQ_N012_ subunit of each dimer is accessible to vHH04 in the dodecameric complex. The relatively low affinity of vHH04 (micromolar range) for XcpQ_N012_ explains, moreover, why it is not able to disrupt the preassembled dodecamer to access the hidden epitope.

10.1128/mBio.01185-17.9 FIG S7  Identification and binding properties of vHH04 nanobodies to XcpQ_N012_. (A) Copurification experiments of purified XcpQ_N012_ with His-tagged vHH04. L, loading material; FT, flowthrough; W1, wash 1; W8, wash 8; E, elution. The presence of untagged proteins in the elution fractions indicates their direct interaction with vHH04. (B) Characterization of vHH04-XcpQ_N012_ binding using biolayer interferometry (BLi). Colored curves represent the association and the dissociation of increasing concentrations of soluble vHH04 (from 0.156 to 10 µM) on XcpQ_N012_ fixed on the streptavidin sensors using biotin tag. The red curves represent the statistical fitting of the experimental curves. vHH04 binds XcpQ_N012_ with micromolar range. (C) SEC profile of purified vHH04 mixed with the isolated XcpQ_N012_ dimers and superimposed with the SEC of purified XcpQ_N012_. Peaks corresponding to the isolated complexes were analyzed by 15% SDS-PAGE–Coomassie blue staining (left insets). L and H indicate the XcpQ_N012_ dimeric and dodecameric complexes. (D) SEC profile of purified vHH04 independently mixed with the oligomeric XcpQ_N012_ (gray line) and superimposed with the SEC of purified XcpQ_N012_ alone (black line). Peaks corresponding to the isolated complexes were analyzed by 15% SDS-PAGE–Coomassie blue staining (left inset). L and H indicate the XcpQ_N012_ dimeric and dodecameric complexes. Molecular mass markers (in kDa) are indicated on the left. Download FIG S7, PDF file, 0.4 MB. Copyright © 2017 Douzi et al. 2017 Douzi et al. This content is distributed under the terms of the Creative Commons Attribution 4.0 International license.

In order to precisely define the vHH04 binding site on its antigen XcpQ_N012_, we solved the crystal structure of the vHH04-XcpQ_N012_ complex to a resolution of 2.9 Å (Table 1). The crystal structure revealed two vHH04s bound at the opposite sides of the XcpQ_N012_ dimer interface (Fig. 2E and F). The vHH04 binding site involved mostly electrostatics and hydrophobic contacts and was limited to the β1 and β3 strands of the N0 subdomain (Table 2). The interface between vHH04 and XcpQ_N012_ buries 510 Å^2^ in agreement with the relatively weak affinity determined by BLi experiments. The crystal structure superimposition of XcpQ_N012_ dimers with or without vHH04 showed that the binding of vHH04 does not induce significant conformational changes in the XcpQ_N012_ dimer (Fig. 2G). This structural information together with previous SEC interference data suggests that the N0 subdomain is involved in the hexamerization of XcpQ_N012_ dimers.

### Atomic model of XcpQ_N012_ dodecamers.

**FIG 4:**
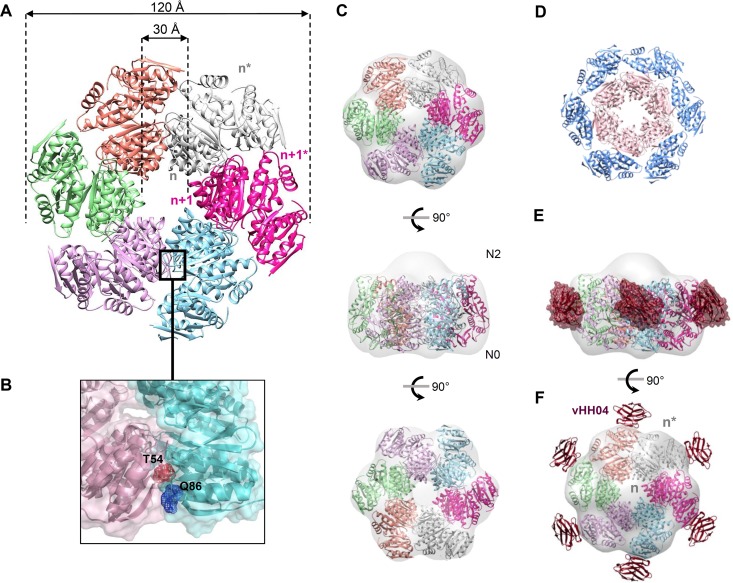
Atomic model of the XcpQ_N012_ dodecameric complex. (A) Model of XcpQ_N012_ dodecamer generated by SymmDock by imposing a C6 symmetry. The six XcpQ_N012_ dimers are shown in cartoon form with a specific color. (B) The residues Thr54 and Gln86 in the N0^*n*+2^-N0^*n*+3^ interface are shown in stick and mesh presentations. (C) Top, side, and bottom views of the dodecameric XcpQ_N012_ model docked into the EM map of the complex. (D) The dodecameric XcpQ_N012_ model is organized into two peripheral and internal rings. The peripheral ring is colored in blue and presents the denoted subunits of each of the six dimers. The inner ring is colored in pink and presents the un-stranded subunits of each of the six dimers. (E) Model of vHH04-XcpQ_N012_ complex. The crystal structure of the vHH04-XcpQ_N012_ complex is overlaid with the dodecameric model of XcpQ_N012_. Only six vHH04s presented in hot pink are able to bind the XcpQ_N012_ complex. (F) Side view of the 6:12 vHH04-XcpQ_N012_ complex docked into the EM map.

Our *in vitro* data revealed that XcpQ_N012_ oligomerizes into a hexamer of dimers. In order to improve our understanding of XcpQ multimerization, we used the SymmDock server (46, 47) to generate a dodecameric complex assuming C6 symmetry by imposing the XcpQ_N012_ dimer as an asymmetric subunit. We kept the best model based on SymmDock criteria and tested it against the numerous constraints imposed by the nanobody binding interface, cysteine cross-linking, and EM map. In the resulting model (Fig. 4A; see Text S2 for coordinates), six XcpQ_N012_ dimers, assembled in a face-to-face manner through their N0-N2 subdomains, interact through their sides, forming a flower-like structure with six petals. The model shows that the XcpQ_N012_ dodecamer is organized into two peripheral and internal rings, where each ring is composed by six XcpQ_N012_ molecules (Fig. 4D). The dodecamer assembly is a consequence of the interaction of the subunits of the internal ring in a lateral fashion, whereas no interaction was established between the subunits of the peripheral ring (Fig. 4D). In this model, the total buried surface between neighboring internal subunits totals 1,400 Å^2^, suggesting that such associations are possible in a physiological environment. Most of the interactions are localized between N0^*n*^-N0^*n*+1^ and N2^*n*^-N2^*n*+1^ with 400 and 460 Å^2^, respectively.

10.1128/mBio.01185-17.2 TEXT S2  Coordinates of the dodecameric model of XcpQ_N012_. Download TEXT S2, TXT file, 1.5 MB. Copyright © 2017 Douzi et al. 2017 Douzi et al. This content is distributed under the terms of the Creative Commons Attribution 4.0 International license.

With an external diameter of 120 Å and an internal cavity with a 30-Å diameter, the XcpQ_N012_ dodecameric model could be easily fitted into the EM map (Fig. 4C), thus supporting the accuracy of the model. The orientation of the XcpQ_N012_ dodecameric model was based on our previous observations of the XcpQN_012_-S210C EM map showing an extra density corresponding to the Trx protein. We therefore propose that the N0 subdomains are positioned at the open side of the EM map (Fig. 4C).

Next, we tried to extend the results obtained for vHH04 showing that it binds the dodecameric complex with a 6:12 stoichiometry. To do this, we fitted the crystal structure of XcpQ_N012_-vHH04 into the model. Interestingly, the six exposed XcpQ_N012_s of the peripheral ring are free to bind six vHH04s, whereas the vHH04 binding site on XcpQ_N012_ of the internal ring overlaps the binding to the adjacent protomer, thus explaining the previously observed binding of only six vHH04s on the preformed XcpQ_N012_ dodecamer (Fig. 4E and F).

In order to validate this atomic model, we checked the predicted 6-fold assembly interface between internal ring subunits by engineering cysteine substitutions within this predicted N0^*n*^-N0^*n*+1^ interface. We thus generated a XcpQ_N012_ variant containing two cysteine substitutions at positions Thr54 and Gln86 which are predicted to be close enough (7 Å) to generate a disulfide bridge under oxidative conditions (Fig. 4B). The resulting XcpQ_N012_-T54C-Q86C construct was purified to homogeneity, and its capacity to multimerize under oxidative conditions was evaluated by SEC and SDS-PAGE. The oxidative condition-dependent oligomerization of the purified XcpQ_N012_-T54C-Q86C from the dodecameric SEC fraction presented in Fig. S4B experimentally demonstrates the close proximity between residues T54 and Q86 in the dodecameric complex. Interestingly, and as predicted from the dodecameric model, the XcpQ_N012_-T54C-Q86C variant is also recovered under higher oligomeric forms such as tetramers and hexamers (Fig. S4B).

### *In vivo* validation of the XcpQ N domain quaternary structure.

**FIG 5:**
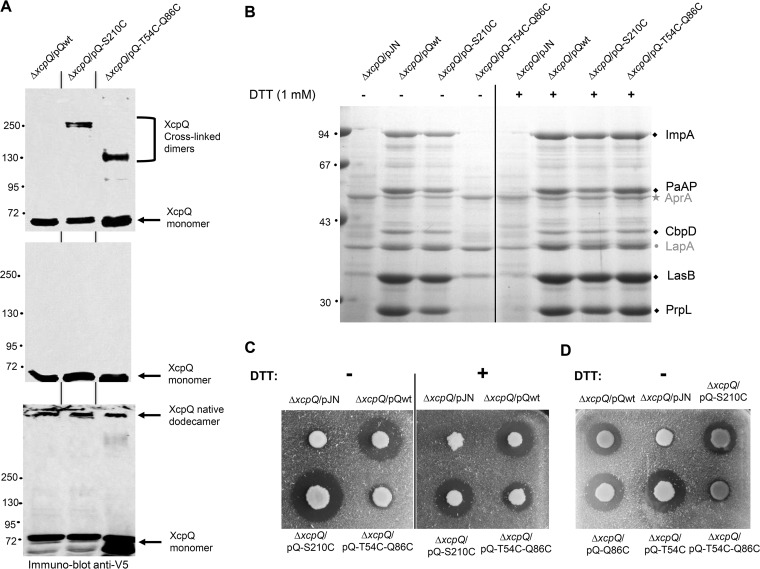
*In vivo* cysteine cross-linking and functionality of full-length XcpQ cysteine variants. (A) Immunoblotting analysis of protein samples from *P. aeruginosa* strains obtained under denaturing and nonreducing (top panel), denaturing and reducing (middle panel), or nondenaturing and reducing (bottom panel) conditions. Molecular mass markers (in kilodaltons) are indicated on the left. (B) Coomassie blue-stained gel of extracellular protein samples from various *P. aeruginosa* strains grown in the presence (+) or not (-) of 1 mM reducing agent dithiothreitol (DTT). The five Xcp T2SS effectors PrpL, elastase LasB, chitin binding protein D (CbpD), aminopeptidase PaAP, and metalloprotease ImpA (43) are indicated by black diamonds; the secreted effectors of the Hxc T2SS alkaline phosphatase LapA (44) and the T1SS alkaline protease AprA (45) are indicated in gray by a dot and a star, respectively. Molecular mass markers (in kilodaltons) are indicated on the left. (C) Extracellular elastase LasB activity of various *P. aeruginosa* strains producing or not producing wild-type or cysteine variants of XcpQ measured on elastin agar plates in the presence (+) or not (-) of 5 mM DTT. The halo of elastin degradation visible around the colony revealed the functionality of the corresponding secretin. (D) Extracellular elastase LasB activity under normal oxidative conditions of various *P. aeruginosa* strains producing or not producing wild-type or cysteine substitutions of XcpQ measured on elastin agar plates. The halo of elastin degradation visible around the XcpQ T54C and Q86C variants revealed their functionality in contrast to the double T54C-Q86C XcpQ variant.

Altogether, our complementary *in vitro* data revealed the quaternary structure of XcpQ_N_ as a hexamer of dimers and allowed us to highlight the residues involved in the assembly. Such bilevel structural organization based on the oligomeric assembly of dimers has already been proposed for full-length secretins (2, 8, 20, 21). In order to investigate the physiological relevance of such structural organization, we tested the ability of the full-length XcpQ secretin to adopt the same fold in its natural environment, the bacterial envelope. We thus tested full-length XcpQ and S210C and T54C-Q86C cysteine substitution variants for their *in vivo* cross-linking and oligomerization capacities by producing them in the *Pseudomonas aeruginosa* PAO1 strain in transcomplementation experiments. Consistent with the previous results obtained by Van der Meeren et al. (8), the membrane protein samples of the wild-type and the XcpQ-S210C cysteine variant analyzed under nonreducing or reducing conditions showed that full-length XcpQ harboring the S210C mutation is able to form stable dimers *in vivo* (Fig. 5A, lane 2). Additionally, the unprecedented analyses of the XcpQ-T54C-Q86C double cysteine variant revealed its ability to form dimers, thus confirming that the proposed interdimeric interface occurs *in vivo* (Fig. 5A, lane 3). The aberrant migration of the XcpQ-S210C dimers during SDS-PAGE is a common behavior for cysteine mutants, especially for membrane proteins (8, 23, 24). Finally, the analysis of the protein samples under reducing but nondenaturing conditions revealed that the two XcpQ variants are also able to assemble secretin channels like the wild-type secretin (Fig. 5A, bottom). Altogether, our *in vivo* data reveal that the atomic organization of the XcpQ_N_ seen *in vitro* also occurs *in vivo*.

### *In vivo* secretin dynamics uncovered by cysteine cross-linking.

We have shown that the XcpQ_N012_ intersubunit interface which supports *in vitro* self-assembly into a hexamer of dimers also occurs *in vivo* in the full-length secretin. This finding indicates that XcpQ secretin adopts, at least temporarily, the conformation seen *in vitro*. Moreover, and as already shown by Van der Meeren et al. (8), we confirmed that the cross-linking of N2/N2* intradimer interfaces at position S210 (Fig. 2B) retains a functional Xcp T2SS, as demonstrated by the extracellular recovery of five Xcp T2SS-secreted effectors (Fig. 5B, left panel; see also Text S1) and the extracellular elastase activity on elastin plates mediated by the Xcp T2SS-secreted effector LasB (Fig. 5C, left panel). In contrast, the nonfunctionality of the XcpQ-T54C-Q86C secretin variant, which locks the N0^*n*^/N0^*n*+1^ interdimers totally, prevents Xcp T2SS secretion in general and LasB activity in particular (Fig. 5B and C, left panels). Of note, in this context the secretion of the 2 Xcp T2SS-independent effectors, AprA and LapA, is not affected, thus indicating a specific inhibition of the Xcp T2SS. We moreover confirmed that T2SS secretion inhibition is specifically due to the T54C-Q86C disulfide bridge and not to any other possible disulfide bridge formed by T54C or Q86C alone, since secretion was not affected in strains producing XcpQ-T54C or XcpQ-Q86C variants (Fig. 5D). Since XcpQ-S210C and XcpQ-T54C-Q86C variants are produced and are able to form disulfide bridges *in vivo* (Fig. 5A), we conclude that, in contrast to the N2/N2* intradimer interface, the lowest part of the secretin, namely, the N0 subdomain, must retain flexibility to allow proper functioning of the XcpQ secretin. This unprecedented notion of a dynamic requirement for secretin function was confirmed by the secretion recovery observed for the XcpQ-T54C-Q86C variant when grown under reducing conditions (Fig. 5B, right panel). Specific secretion of the five Xcp T2SS effectors is recovered in the XcpQ-T54C-Q86C-producing strain when grown in the presence of 1 mM reducing agent dithiothreitol (DTT). In agreement with this result, extracellular elastase activity was also partially recovered under 5 mM DTT reducing conditions for the strain producing XcpQ-T54C-Q86C, thus confirming the reversion of the Xcp T2SS secretion-proficient phenotype (Fig. 5C, right panel). This indicates that disruption of the artificial corresponding disulfide bridge restores secretin functionality, therefore demonstrating the requirement for dynamic secretin activity.

### *In vivo* secretin interactome refined by vHH interference.

**FIG 6:**
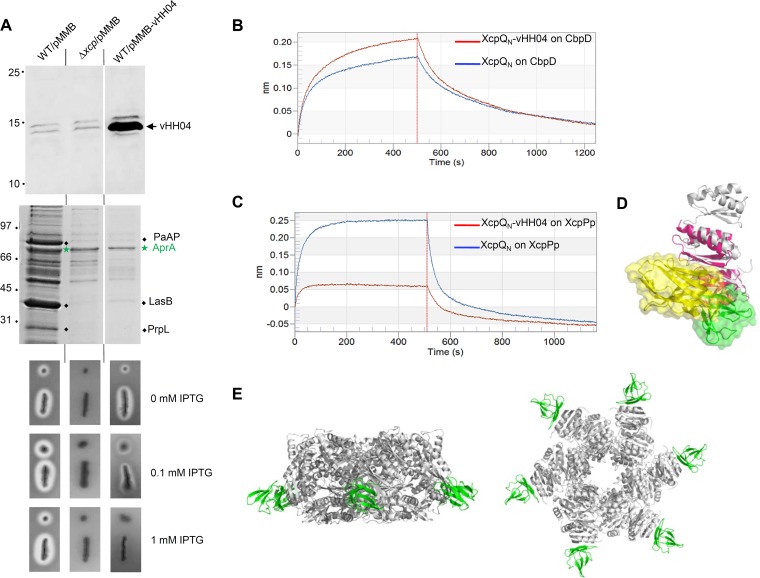
*In vivo* vHH04 production and interference. (A) Immunoblot detection with antihistidine antibody of the histidine-tagged vHH04 in the cellular samples (top panel) or T2SS effector identification on Coomassie blue-stained SDS-PAGE of supernatant protein samples (middle panel) from *P. aeruginosa* strains producing or not producing vHH04. When produced, vHH04 inhibits the specific secretion of the T2SS effectors PaAP, LasB, and the protease PrpL (black diamonds) but not the T1SS effector AprA (green star). The bottom panel shows elastase activity assay on *P. aeruginosa* strains producing or not producing vHH04. The vHH04-mediated interference with Xcp T2SS extracellular protease activity is characterized by the isopropyl-β-d-thiogalactopyranoside (IPTG)-dependent inhibition of the extracellular halo of protease degradation around the colony on skim milk plates. Molecular mass markers (in kilodaltons) are indicated on the left. (B and C) Bilayer interferometry recordings representing binding of XcpQ_N012_ alone (blue) or the XcpQ_N012_-vHH04 complex (red) to a sensor coupled to CbpD (B) or to XcpPp (C). The response (in nanometers) is plotted versus the time (in seconds). The response measured in the red sensorgram (B) shows that vHH04 does not interfere with the binding of XcpQ_N012_ to CbpD, whereas the absence of response in the red sensorgram (C) revealed that vHH04 prevents the binding of XcpQ_N012_ to XcpPp. (D) Superimposition of GspC-GspD complex (PDB ID 3OSS) with the vHH04-XcpQ structure revealing that the vHH04 binding site overlaps GspC (HR) binding to GspD. The structures of XcpQ_N012_, vHH04, GspC (HR), and GspD_N01_ are colored in gray, yellow, green, and hot pink, respectively. (E) Side and bottom views of the XcpPp-XcpQ_N012_ assembly model where only 6 XcpPps (green structures) are bound to the XcpQ_N012_ dodecameric complex (gray structure).

In order to test if the vHH04 nanobody interferes with XcpQ function during type II secretion, we produced it in the periplasm of the wild-type PAO1 *P. aeruginosa* strain (Fig. 6A, top panel). We then tested the functionality of the Xcp T2SS upon vHH04 production by analyzing the supernatant content. As indicated in Fig. 6A (middle panel), the supernatant profile of the wild-type strain producing vHH04 is similar to that of an *xcpQ* deletion mutant, thus indicating the specific dominant negative effect of vHH04 on Xcp T2SS secretion. Xcp T2SS inhibition by vHH04 was confirmed by the gradual inhibition of the elastase activity on skim milk plates upon the gradual induction of vHH04 production (Fig. 6A, bottom panel). We therefore propose that, under those experimental inducible conditions, the nanobody reaches its secretin target and interferes with the essential binding of natural secretin interactants, thus compromising the secretion process.

We and others have shown that T2SS secretins interact, via their N domains, with at least three partners during the secretion process: the secreted effectors, the inner membrane connector protein XcpP, and the pseudopilus tip (7, 12, 14, 24). To test the effect of vHH04 on individual XcpQ-partner interactions, we performed BLi competition experiments with purified soluble XcpP periplasmic domain (XcpPp) and the T2SS effector CbpD. BLi data revealed that the presence of vHH04 does not alter the affinity of XcpQ_N_ for the secreted CbpD (Fig. 6B), thus indicating two different binding sites for vHH04 and CbpD on the secretin. Concerning the well-described interaction between the secretin XcpQ and its inner membrane partner XcpP (14), our BLi data indicated a strong inhibitory effect of this interaction in the presence of purified vHH04 (Fig. 6C), suggesting a possible competition for XcpP and vHH04 binding on XcpQ.

## DISCUSSION

The assembly of secretins into a functional channel was proposed to be driven by the C domain since its purification from different systems leads to oligomeric ring-shaped channels (6, 25). This was, moreover, supported by the structural data on N domains revealing monomeric or dimeric forms, thus assuming that their final oligomeric organization into periplasmic cavity is assisted by the C domain. In the present study, we show that the purified N domain of the *P. aeruginosa* XcpQ T2SS secretin (XcpQ_N012_) spontaneously forms dodecameric complexes organized into a hexamer of dimers. This intrinsic property of the secretin N domain to self-oligomerize shows that the C domain is not absolutely required for this oligomerization process but also attributes to this domain a possible role in secretin folding or biogenesis. According to this, a study suggests that a large number of secretins adopt a prepore structure in the periplasmic space independently from any known insertase machineries before their insertion into the outer membrane (26). These observations are moreover supported by the recent cryo-electron microscopy (cryo-EM) structures of three secretins, proposing for all of them a limited outer membrane insertion domain through an extreme C-terminal short domain while the majority of the protein, including most of the C domain, is localized in the periplasm (3, 4). The same studies revealed that the N3 subdomain, which is common to all secretins, structurally belongs to the C domain. It was moreover shown that the N2 domain is linked to the N3 domain through a long linker loop inducing considerable flexibility between N0-N1-N2 subdomains and the upper N3/C domain. This bipartite structural organization is supported by previous studies showing that the N3 subdomain plays an important role in secretin prepore stabilization by forming a thermodynamic seal (26). Together, these observations combined with our results suggest that T2SS secretins are structurally organized into two structurally independent domains, the N domain, composed of N0, N1, and N2 subdomains, and the C domain, constituted by the rest of the protein (N3 and former C domain).

The natural propensity of the N domain to spontaneously assemble into homodimers under a C2 symmetry has already been reported for XcpQ (8) and other secretins such as OutD from *Dikeya dadantii* (24) and HofQ from *Aggregatibacter actinomycetemcomitans* (27). This observation logically interrogates the structural organization of the C-terminal domain which, in contrast, adopts a C1 symmetry (3, 4). Such a discrepancy remains nevertheless compatible, owing to the flexible connection that exists between the N and C domains as reported above in the discussion and shown by the low-pass-filtered EM map recovered between the N2 and N3 rings, which leave enough space to accommodate such structural reorganization (3).

Our data revealed a dodecameric assembly of the XcpQ secretin N-terminal domain, an oligomerization order also found in other secretins such as PulD from T2SS of *Klebsiella oxytoca* (6), PilQ of T4P from *Neisseria meningitidis* (28), and PscC of T3SS from *P. aeruginosa* (6). Recent high-resolution cryo-EM structures of T2SS and T3SS secretins unambiguously revealed a C15-fold symmetry throughout their C domains (3, 4). As clearly presented in the accompanying paper (29), such C15-fold symmetry is also recovered for the T2SS secretin XcpQ, thus revealing that for a dedicated secretin, different stoichiometries can be adopted. Such variability in secretin’s symmetries raises the question of the dynamics of assembly of these channels, and as suggested by Hay et al. in the accompanying paper (29), the different stoichiometries can be triggered by the presence of secreted effectors and/or other interacting partners in order to achieve their function. Following this idea and in agreement with our data, Hay et al. are proposing in the accompanying paper (29) a reconciling model where XcpQ transits from C15 to C6 symmetry via a radially symmetric array wherein three N domains are displaced to create a metastable arrangement compatible with the 6-fold symmetry structures proposed for the inner membrane assembly platform (see Fig. 2E of reference 29).

During secretion, the N domain of the secretin must accommodate large protein complexes with diameters of 60 to 80 Å. The XcpQ secretin N domain purified in this study presents constrictions of 30 Å wide at both extremities which are incompatible with substrate passage, thus suggesting a closed conformation. This domain must therefore undergo significant structural rearrangements during the secretion process, possibly through intrinsic-disorder domains as proposed by Gu et al. (30). Such dynamic behavior is supported by our *in vivo* cysteine cross-linking experiments showing that locking the N0^*n*^-N0^*n*+1^ interface (Fig. 4B and 5) has a drastic effect on the T2SS function. Further experiments performed under reducing conditions revealed that secretin functionality is fully recovered upon reduction of the artificial disulfide bridge. This locking-mediated loss of activity indicates that the secretin channel requires a high degree of flexibility within the N0 ring to be functional. Such flexibility was presaged by the absence or the smeared densities of the N0 ring reported in the cryo-EM secretin structures (3, 4, 29). In contrast, the functionality of the XcpQ-S210C variant that freezes the N2-N2* interface suggests that secretin function does not require flexibility within the N2 ring. All these results suggest that the XcpQ N domain oligomer isolated *in vitro* represents the resting closed state of the secretin that upon substrate arrival may switch to an open state. Such switching was supported by our *in vivo* experiments indicating that the locking of the closed state led to a nonfunctional secretin, which regained functionality when we reduced the disulfide bridge. Such switching from a closed to an open state has already been proposed for T4P secretins. In fact, the electron cryotomography (ECT) structures revealed that in the absence of their dedicated pili, the secretins present an additional periplasmic gate holding the N domain in a closed state (30). The presence of this gate has never been observed in purified secretins, suggesting that this is a highly dynamic phenomenon occurring in the native environment. More generally, dynamism in outer membrane gates of bacterial secretory machines has also been proposed for the TssJLM transmembrane complex of the type VI secretion systems (31). The authors have proposed that the TssJLM complex transits from a C5-symmetry closed conformation to a C10-symmetry open conformation to allow the injection of the dedicated effectors in the recipient cells.

Our vHH04 interference data on secretin function and secretin interaction with different partners allowed us to propose two major improvements in the global understanding of the T2SS architecture. Considering the vHH04 binding site on the secretin and its properties of interference with XcpP binding, we are proposing a similar positioning for XcpP at the periphery of the XcpQ_N01_ subdomain (Fig. 6D and E). Such positioning is in full agreement with the previous X-ray 3D costructure determination of the GspC-GspD complex revealing that GspC, the XcpP homolog in the *E. coli* T2SS, interacts with its secretin GspD in a region closed to the vHH04 binding site (Fig. 6D) (14). Taking into account the structural organization of the secretin N domain and the XcpP binding site, we strikingly observed that, similarly to vHH04, only six XcpPs can access the XcpQ_N_ dodecamer, while the six other potential XcpP binding sites are involved in N0^*n*^-N0^*n*+1^ interactions (Fig. 6E). Considering that GspE ATPase forms hexamers in the cytoplasmic space, it was proposed that the transenvelope complex of GspE/L/M/C is assembled through cyclic C6 symmetry (32–35). Thus, we propose that the transition from 12- to 6-symmetry order between the outer membrane and the cytoplasmic space takes place between GspD and GspC (Fig. 6E).

One intriguing feature of the T2SS is its ability to secrete a wide range of effectors under their folded states. This function implies a specific recognition between the secretion system components and secreted effectors. We previously showed specific interactions between the XcpQ_N01_ subdomains and the effector LasB (12). Interestingly, we demonstrate in this study that the recognition domain of the effectors on the N domain may differ from the GspC binding site, an observation in agreement with the passage of the effector through the cavity of the N domain.

Taken together, our data provide significant insights into the understanding of secretin organization, functioning, and interactome in the context of type II secretion. They moreover provide new tools and open new directions for further investigations, necessary to fully understand the dynamics and the assembly process of T2SS secretins, the role of the interacting partners in secretin dynamics, and the effect of IM partners on secretin function.

## MATERIALS AND METHODS

See Text S1 and Table S1 in the supplemental material for additional details regarding the methods.

10.1128/mBio.01185-17.10 TABLE S1  Plasmids and oligonucleotides used in this study. Download TABLE S1, DOCX file, 0.02 MB. Copyright © 2017 Douzi et al. 2017 Douzi et al. This content is distributed under the terms of the Creative Commons Attribution 4.0 International license.

### Bacterial strains.

The *Escherichia coli* K-12 DH5α (laboratory collection), WK6 (22), and BL21(DE3)pLysS (laboratory collection) strains were used for cloning procedures, nanobodies, and soluble protein production, respectively. *Pseudomonas aeruginosa* PAO1 wild-type (laboratory collection), PAO1 *ΔxcpQ* (36), and PAO1 *Δxcp* (also called D40ZQ) (37) strains were used for nanobody interference and *in vivo* cysteine cross-linking.

### Crystallization of XcpQ_N012_ and the complex XcpQ_N012_-vHH04.

Crystallization assays of XcpQ_N012_ and the XcpQ_N012_-vHH04 complex have been undertaken using various crystallization kits such as Stura, Wizard, MDL, JCSG+, Index, PEG I, Proplex, Cations, Anions, and Additives, using the sitting-drop vapor-diffusion method at 20°C and 4°C. Crystallization hits of XcpQ_N012_ (20 mg/ml) were obtained with a variety of polyethylene glycol (PEG) conditions yielding different crystal shapes, mostly very thin needles. After several optimization rounds, the final crystallization conditions were 0.8 M lithium chloride, 0.1 M Tris-HCl (pH 8.5), 0.1 M sodium acetate, and 32% (wt/vol) PEG 4000. The XcpQ_N012_-vHH04 complex (6 mg/ml) was crystallized in 0.1 M bis-Tris (pH 5.5), 25% (wt/vol) PEG 3350. Crystals appeared after 14 days at 20°C.

### Structure determination of XcpQ_N012_ and the complex XcpQ_N012_-vHH04.

Crystals of XcpQ_N012_ were briefly transferred in mother crystallization solution supplemented with 10% (vol/vol) PEG 4000, while the crystals of the XcpQ_N012_-vHH04 complex were soaked in their native reservoir solution containing 25% (wt/vol) PEG 3350. The crystals were flash-cooled in a nitrogen gas stream at 100 K. The X-ray diffraction data for XcpQ_N012_ and the complex XcpQ_N012_-vHH04 were collected at Proxima 1 and Proxima 2 (Soleil synchrotron, Saint-Aubin, France), respectively. The diffraction images were integrated, scaled, and merged with the XDS package (38) (Table 1).

The crystal of native XcpQ_N012_ and the complex XcpQ_N012_-vHH04 diffracted to 2.98 Å and 2.9 Å, respectively. The space group of the XcpQ_N012_ crystal is P2_1_, with the following cell dimensions: *a* = 40.4, *b* = 122.25, and *c* = 55.44 Å and α = 90°, β = 109.06°, and γ = 90°. Crystals of the XcpQ_N012_-vHH04 complex belong to the triclinic space group P1, with the following cell dimensions: *a* = 40.06, *b* = 63.79, and *c* = 76.05 Å and α = 104.37°, β = 100.61°, and γ = 108.04°. Molecular replacement was performed in both cases using the published structure of XcpQ (100% sequence identity, PDB accession no. 4E9J) and the structure of a nanobody with high sequence similarity (PDB accession no. 5M2W) (39). The model was improved by alternated cycles of autoBUSTER (40) refinement and manual building with Coot (41). Data and refinement statistics are provided in Table 1. The interaction contacts between the dimeric XcpQ_N012_ and vHH04 were analyzed using the protein interfaces, surfaces, and assemblies service of the Protein Data Bank (PDBe PISA) (42).

### Negative-staining single-particle analysis electron microscopy and 3D model reconstitution.

For the XcpQ_N012_ complex, 10-μl drops of the suitably diluted (0.02 mg/ml) sample suspension were placed directly on glow-discharged carbon-coated copper grids (Electron Microscopy Sciences [EMS]) for 2 min. The grids then were washed with 2 drops of 2% aqueous uranyl acetate and stained with a third drop for 1 min. Images were recorded on an FEI Tecnai 200-kV electron microscope operating at a voltage of 200 kV and a defocus range of 0.5 to 2.5 µm, using an Eagle charge-coupled device (CCD) camera (FEI) at a nominal magnification of ×50,000, yielding a pixel size of 4.4 Å. A total of 500 particles were automatically selected from 60 independent images and extracted within boxes of 100 by 100 pixels using EMAN2. The defocus value was estimated, and the contrast transfer function was corrected by phase flipping without further corrections using EMAN2 (e2ctf). All 2D classifications were performed using EMAN2. We used three rounds of reference-free 2D class averaging to clean up the automatically selected data set.

For Trx-XcpQ_N012_ and Trx-XcpQ_N012_-S210C complexes, 10 μl of suitably diluted (0.05 mg/ml) Trx-XcpQ_N012_ complex and 0.02 mg/ml of Trx-XcpQ_N012_-S210C samples were treated as described above for XcpQ_N012_. A total of 1,660 particles for Trx-XcpQ_N012_ and 3,600 particles for Trx-XcpQ_N012_-S210C were automatically selected from 118 and 103 independent images, respectively, and extracted within boxes of 100 by 100 pixels using EMAN2. The defocus value was estimated, and the contrast transfer function was corrected by phase flipping without further corrections using EMAN2 (e2ctf). All 2D and 3D classifications and refinements were performed using EMAN2. An initial 3D model was generated in EMAN2 using 15 and 25 classes for Trx-XcpQ_N012_ and Trx-XcpQ_N012_-S210C, respectively. All particles were used for refinement. Based on biochemical data suggesting that XcpQ_N012_ assembles into dimers and dodecamers, we applied C6 and C12 symmetries. The 6-fold symmetry axis offered the sharpest resolution. The EMAN2 autorefine procedure was used to obtain a final reconstruction at 31-Å and 25-Å resolutions after masking and with C6 symmetry imposed for Trx-XcpQ_N012_ and Trx-XcpQ_N012_-S210C.

### *In vitro* cysteine cross-linking.

The XcpQN_012_-S210C and XcpQ_N012_-T54C-Q86C variants were purified to homogeneity according to the same protocol used for XcpQ_N012_. For the XcpQN_012_-S210C variant, after the tobacco etch virus (TEV) cleavage step and the nickel affinity chromatography purification steps, the protein was concentrated to 20 mg/ml using a 10K Centricon (Millipore). Next, the protein was incubated with 0.3% (vol/vol) H_2_O_2_ for 30 min at room temperature. Then, 5 ml of the treated sample was subjected to SEC purification using a HiLoad Superdex 200 16/600 column preequilibrated on 50 mM Tris-HCl (pH 8) and 150 mM NaCl. Seventy-five microliters of the fractions corresponding to XcpQ_N012_ dodecameric complex was mixed with 25 µl of Laemmli loading buffer (125 mM Tris-HCl, pH 6.8, 0.002% bromophenol blue, 2% SDS, and 10% glycerol) with or without 20 mM β-mercaptoethanol (β-ME) for reducing or nonreducing conditions, respectively. The samples were heated for 5 min at 95°C and then loaded into 12% SDS-PAGE gels.

For the XcpQ_N012_-T54C-Q86C variant, the H_2_O_2_ treatment leads to large protein precipitation. Suspecting that this precipitation was induced by disulfide bridge formation, we bypassed the oxidation step and subjected this variant to SEC purification without H_2_O_2_ treatment. The fractions of the HilLoad Superdex 200 16/600 column corresponding to the oligomeric form of XcpQ_N012_-T54C-Q86C were analyzed under reducing and nonreducing conditions as described for the XcpQN_012_-S210C variant.

### XcpQ cysteine variant production in *P. aeruginosa.*

Expression of the different constructs was performed in *P. aeruginosa*. *P. aeruginosa* strains (PAO1*ΔxcpQ/pJN*, PAO1*ΔxcpQ/pJN-XcpQ*, PAO1*ΔxcpQ/pJN-XcpQ-S210C*, and PAO1 *ΔxcpQ/pJN-XcpQ-T54C-Q86C*) were grown overnight at 37°C in LB liquid medium supplemented with 50 µg/ml of gentamicin. These cultures were used to inoculate 25-ml cultures in tryptic soy broth (TSB) liquid medium at an optical density at 600 nm (OD_600_) of 0.1. When OD_600_ reached 0.8, 0.1% filtered l-arabinose was added to induce protein expression from the pJN vector. After 3 h of induction at 37°C, the equivalent of 5 OD_600_ units was centrifuged. The pellet was resuspended in 1 ml of 50 mM Tris-HCl (pH 8.0), 150 mM NaCl, 1% IGEPAL, 10 mM EDTA, and 5 mM *N*-ethylmaleimide to block any available cysteine sulfhydryls supplemented with a mixture of protease inhibitors (Complete; Roche). The cells were disrupted by 4 short cycles of sonication and incubated on ice for 30 min. Cell membrane was recovered by centrifugation at 20,000 × *g* for 30 min and then dissolved in 200 µl of Laemmli loading buffer (2% SDS [wt/vol], 10% glycerol [vol/vol], 0.002% [wt/vol] bromphenol blue, and 125 mM Tris-HCl, pH 6.8) and heated for 10 min at 95°C. After 5 min, 10 µl of β-mercaptoethanol (β-ME) or distilled H_2_O was added to 90 µl of sample and heated again for 5 min at 95°C. Finally, 15 µl was loaded on a 4 to 15% SDS-PAGE gel and the presence of XcpQ protein harboring a V5-C-terminal tag was checked by Western blotting using anti-V5 (Bethyl) antibodies at 1/5,000.
