# Supplementary material for: Unraveling the Self-Assembly of the Pseudomonas aeruginosa XcpQ Secretin Periplasmic Domain Provides New Molecular Insights into Type II Secretion System Secreton Architecture and Dynamics
Source: mBio. 2017 Oct 17;8(5):e01185-17. doi: 10.1128/mBio.01185-17 (PMC5646246; doi:10.1128/mBio.01185-17)
Supplement: TEXT S1 [file mbo005173532s1.docx]

**SUPPLEMENTARY METHODS**

**Plasmid construction for *in vitro* and *in vivo* studies**

Plasmids used in this study are listed in **Table S1A**. Polymerase Chain Reactions (PCR) were performed using the Q5 high fidelity DNA polymerase (New England BioLabs) for routine cloning or Pfu Turbo (Agilent) for quick-change mutagenesis. Custom oligonucleotides, listed in **Table S1B**, were synthesized by IDT Integrated DNA Technologies (IDT). *P. aeruginosa* chromosomal DNA was used as a template for all PRCs. Genes encoding the N-terminal domains N0N1 (residues 35-207) and N0N1N2 (residues 35-280) of XcpQ as well as the periplasmic domain of XcpP (XcpPp) (residues 56-235) were cloned into pETG-20A expression vector using Gateway technology (1). The gene encoding for the XcpQ full-length protein including their proper signal peptides were cloned into pJN105 vector using SLIC technology (Jeong et al, 2012). To facilitate XcpQ detection on western blot experiment, a V5-tag was added at the C-terminus by PCR. The gene encoding for CbpD effector including its proper signal peptide was sub-cloned from pCbpD to pET-DUET (Invitrogen) by restriction using EcoRI. The gene encoding vHH04 was sub-cloned from pHEN6 vectors to pMMB67HE vectors by restriction using EcoRI and HindIII. To construct cysteine variants, the pJN105-XcpQ and pETG-20A-XcpQ_N012_ were used as template. Mutagenesis was performed using quick change technology (Stratagen). The two complementary primers (see **Table S1B**) containing the desired mutation were used to amplify the entire plasmid by invers PCR. When necessary, the plasmid harboring the cysteine codon mutation was used for a second round to insert the second cysteine codon mutation. All constructs have been verified by restriction analysis and DNA sequencing (GATC).

**Protein production and purification**

The periplasmic domains of the XcpQ and XcpP proteins corresponding to XcpQ_N01_, XcpQ_N012_ and XcpP_p_ were produced and purified as described in (1) with slight modifications. All XcpQ and XcpP variants were produced in presence of a cleavable N-terminal thioredoxin (Trx) extension to increased cytoplasmic stability. Briefly, BL21(DE3) competent cells were transformed by the pETG-20A-XcpQ_N01_, pETG-20A-XcpQ_N012_ and pETG-20A-XcpP_p_. The transformed cells were plated on lysogeny broth (LB) agar plates containing 100 mg/L ampicillin (LB^amp^). Several colonies were picked and inoculated into 100 ml LB^amp^. The cultures were grown with shaking at 37°C overnight. Overnight cultures were dispersed as inoculum of 25 ml aliquots into 1.25 L of Terrific broth (TB) media in the presence of 100 mg/L of ampicillin (TB^amp^) and shaken at 37°C until the OD_600_ reached 0.5 unit, then temperature was dropped to 25°C and cells were induced with 0.5 mM IPTG and for an overnight growth at 25°C. Cells were centrifuged at 9,000 x g for 10 minutes at 4°C. Dry cell pellet was stored at -80°C. For protein purification, cell pellets were thawed at room temperature for 20 min and resuspended in lysis buffer composed by buffer A (50 mM Tris-HCl pH 8.0, 300 mM NaCl, 10 mM imidazole) supplemented with 1 mM EDTA, 0.5 mg/ml lysozyme, 1 mM phenylmethylsulfonyl fluoride, submitted to several freeze-thawing cycles and sonicated after the addition of 20 µg/ml DNase and 20 mM MgCl_2_. Pellet and soluble fractions were separated by centrifugation for 30 min at 16,000 x g. The soluble fraction containing Trx-XcpQ_N01_, Trx-XcpQ_N012_ and Trx-XcpP_p_ proteins were loaded onto a 5-ml Nickel column (HisTrap™ FF) using an ÄKTA prime apparatus (GE healthcare) and the immobilized proteins were eluted in buffer B (50 mM Tris-HCl pH 8.0, 300 mM NaCl, 250 mM imidazole). XcpQ_N01_, XcpQ_N012_ or XcpP_p_ were obtained after cleavage of the Trx fusion using 2 mg of TEV protease for 18 hours at 4°C on a dialysis bag allowing imidazole removal. Untagged soluble proteins were then collected in the flow-through of a 5-ml Nickel column while the histidine-tagged TEV and Trx proteins remain bound to the column. The proteins were concentrated using the centricon technology (Millipore, 10-kDa cut-off) and subjected to size exclusion chromatography (SEC) purification using a HiLoad Superdex200 16600 column pre-equilibrated with 50 mM Tris-HCl pH 8.0, 150 mM NaCl for gel filtration chromatography.

For CbpD effector, the gene encoding CbpD including its proper signal peptide and a His_6_-tag at its C-terminus was cloned into pET-DUET vector. *E. coli* BL21(DE3) cells cultivated in the TB medium carrying plasmid pET-DUET-CbpD were grown to OD_600_ of 0.6 unit and the expression of CbpD was induced by the addition of 0.5 mM IPTG for 16 h at 28°C. Cells were collected by centrifugation at 10,000 g at 4°C for 15min. The cell pellet was resuspended in cold TES buffer (200 mM Tris-HCl pH 8.0, 0.5 mM EDTA and 0.5 M Sucrose) and kept on ice for 1h. The periplasmic proteins were then removed by osmotic shock by addition of TES buffer diluted to 1/4 in cold H_2_O. After 2 h incubation on ice, the suspension was centrifuged at 20,000 g for 30 min. The supernatant was loaded into 5-ml Nickel column pre-equilibrated on buffer A. Soluble CbpD was eluted in buffer B and concentrated using the centricon technology with 10K cut-off. After concentration step, CbpD was subjected to SEC purification using a HiLoad Superdex200 16600 column pre-equilibrated with 50 mM Tris-Hcl pH 8.0, 150 mM NaCl for gel filtration chromatography.

**Generation of camel nanobodies against XcpQ N-terminal domain**:

Purified XcpQ_N_ was produced and purified as previously described (1). Six injections of 0.8 mg of recombinant XcpQ_N_ in Tris-HCl 20 mM pH 8.0, NaCl 150 mM were performed subcutaneously with one week intervals in a dromadery (*Camelus dromedarius*) from the Canaria Islands. Lymphocytes were isolated from blood sample obtained 1 week after the last immunization. cDNA was synthesized from purified total RNA by reverse transcription. The cDNA was used as template for PCR amplification to amplify sequences corresponding to the variables domains of the heavy-chain antibodies. PCR fragments were cloned into the phagemid vector pHEN4 to create a nanobody phage display library. Selection and screening of nanobodies were performed as previously published (2). Three rounds of panning resulted in the isolation of XcpQ_N_-specific binders. Nanobody vHH04 was selected, sequenced and cloned into the pHEN6 expression vector downstream the pelB signal peptide and fused to a C-terminal 6×His tag for periplasmic production in *E. coli* and further subcloned into pMMB67HE vector for periplasmic production in *P. aeruginosa*.

**vHH04 production and Purification**

For vHH04 production and purification, *E. coli* WK6 cells carrying the pHEN6-vHH04 were grown at 37°C in TB medium to an optical density OD_600_ of 0.8 unit. The expression of the nanobody was induced by the addition of 1 mM IPTG and incubation for 16 hours at 28°C. The periplasmic fraction containing the nanobodies was prepared by osmotic shock as described previously (see Protein production and purification section). The His-tail-containing fusion proteins were purified by immobilized metal affinity chromatography on a 5-ml Ni-NTA column equilibrated in buffer A. vHH04 was eluted in buffer B and concentrated (Amicon- Ultra 10-kDa cut-off) prior being subjected to SEC purification using a HiLoad Superdex200 16600 gel filtration column equilibrated in 50 mM Tris–HCl pH 8.0, 150 mM NaCl.

**Affinity chromatography on magnetic nickel beads**

We used as bait 100 µg of his_6_-tagged vHH04. All experiments were carried out at 4°C. In the reaction mixture 1 (RM1), vHH04 was incubated in 1 ml of Equilibration Buffer (EqB) (50 mM Tris-HCl pH 8.0, 300 mM NaCl, 10 mM Imidazole) with 200 μl of 5% Ni-NTA magnetic-beads solution (Millipore) pre-equilibrated in EqB. In the reaction mixture 2 (RM2), the untagged XcpQ_N012_, was incubated in 1 ml of EqB. Both RM1 and RM2 were placed on a rotary shaker and gently mixed for 1 h. RM1 was placed on a magnet rack (Millipore) for 1 min in order to catch the magnetic-beads. The flow-through (FT1) was discarded and the magnetic-beads rinsed twice with 500 μl of EqB (W1 and W2), including for each wash 1 min mixing on the rotary shaker and 1 min catch on the magnet. Then, RM2 was mixed with the vHH04-coated magnetic-beads issued from RM1 and gently mixed on the rotary shaker for 1 hour. After 1 min catch on the magnet, the flow-through (FT2) was discarded and the magnetic-beads were washed with 500 μl of wash buffer (WB) (50 mM Tris-HCl pH 8.0, 300 mM NaCl, 20 mM Imidazole) six times (W3-W8), including for each wash 1 min mixing on the rotary shaker and 1 min catch on the magnet. Proteins specifically bound to the magnetic-beads were then eluted with 100 μl of elution buffer (EB) (50 mM Tris-HCl pH 8.0, 300 mM NaCl, 500 mM Imidazole) two times (E1-E2), including for each elution 1 min mixing on the rotary shaker, a spin at 8,734 x g to pellet the beads and 1 min catch on the magnet.

**Bio-layer Interferometry**

XcpQ_N012_, XcpP_p_ and CbpD were biotinylated using the EZ-Link NHS-PEG4-Biotin kit (Perbio Science, France). The reaction was stopped by removing the excess of the biotin using a Zeba Spin Desalting column (Perbio Science, France). BLi studies were performed in black 96-well plates (Greiner) at 25°C using an OctetRed96 (ForteBio, USA). Streptavidin biosensor tips (ForteBio, USA) were first hydrated with 0.2 ml of interaction buffer (IB) (50 mM Tris-HCl pH8, 150 mM NaCl, 0.1% BSA and 0.1%vTween) for 20 min and then loaded with biotinylated proteins (10 μg/ml in IB). For the association of vHH04 with XcpQ_N012_, increasing concentrations of vHH04 (0.156 to 10 µM) were used and the association phases were monitored for 600sec. To test the interference of vHH04 on XcpQ_N012_/XcpP_p_ or XcpQ_N012_/CbpD complexes, a mixture of XcpQ_N012_:vHH04 with a ratio of 1:1.2 or free XcpQ_N012_ were tested for their ability to bind XcpPp or CbpD. The association and dissociation phases were monitored for 500 and at least 600 sec, respectively. In all experiments, the response of the non-biotinylated proteins on the free sensors was subtracted during experiment processing.

**Nanobodies production in *P. aeruginosa***

*P. aeruginosa* PAO1 strains harboring plasmid pMMB67HE-vHH04 was grown at 37°C in LB liquid medium overnight. The O/N pre-culture was used to inoculate 100 ml of LB^amp^ to OD_600_ of 0.1 unit. After 3 hours of growth at 37°C, vHH04 production was induced by the addition of 1 mM IPTG. After 2 hours, cell cultures were centrifuged to separate the cell pellet from the supernatant. The cell pellets were treated by benzonase (1/100) and Laemmli loading buffer (1X) and heated at 95°C for 5 min. The vHH04 production was then analyzed on a 12% sodium dodecyl sulfate-polyacrylamide gel stained by Coomassie blue.

**Analysis of protein samples**

Purified, total cell, membrane or supernatant protein samples obtained following the previously described procedure for purified proteins or as described in (3) for cellular derived-fractions were analyzed under denaturing (SDS-PAGE) or non-denaturing (ND-PAGE) polyacrylamide gel electrophoresis conditions are described in (4) or by blue native (BN-PAGE) condition as described in (5). When indicated, PAGE were immuno-blotted as described in (4) using mouse anti-His at 1/1000 (Qiagen) or rabbit anti-V5 antibodies.

**Elastase and protease activities on plate.**

Elastase activity was tested by spotting (2 µl) bacteria on tryptic soy agar (TSA) plate containing 1% elastin and Gentamycin at 20 µg/ml followed by 4 days incubation at 30°C. Protease activity was tested by streaking bacteria on TSA plates containing 1.5% lyophilized milk, Carbenicillin at 100 µg/ml and IPTG at the indicated concentration followed by 2 days incubation at 30°C.

**Analytical UltraCentrifugation (AUC).**

Sedimentation velocity experiments were carried out at 40000 rpm and 20°C in a Beckman Optima-XL-A analytical ultracentrifuge, using 3 mm double sector centerpieces in an AN50Ti rotor. Scans were acquired in continuous mode at 280 nm, in the range of 0.1 to 1 absorption. At 20°C, the partial specific volume of XcpQ_N012_, solvent density and viscosity calculated with SEDNTERP (6), were 0.73806 ml/g, 1.00585 g/cm3 and 0.01002 poise respectively. The data recorded from moving boundaries were analyzed in terms of both discrete species and continuous size distribution functions of sedimentation coefficient, C(S) using the program SEDFIT (7).

**References**

1. **Douzi B, Ball G, Cambillau C, Tegoni M, Voulhoux R.** 2011. Deciphering the Xcp Pseudomonas aeruginosa type II secretion machinery through multiple interactions with substrates. J Biol Chem **286:**40792-40801.

2. **Desmyter A, Farenc C, Mahony J, Spinelli S, Bebeacua C, Blangy S, Veesler D, van Sinderen D, Cambillau C.** 2013. Viral infection modulation and neutralization by camelid nanobodies. Proc Natl Acad Sci U S A **110:**E1371-1379.

3. **Ize B, Viarre V, Voulhoux R.** 2014. Cell fractionation. Methods Mol Biol **1149**:185-191

4. **Viarre V, Cascales E, Ball G, Michel GP, Filloux A, Voulhoux R.** 2009. HxcQ liposecretin is self-piloted to the outer membrane by its N-terminal lipid anchor. J Biol Chem **284:**33815-33823.

5. **Dalal K, Duong F.** 2010. Reconstitution of the SecY translocon in nanodiscs. Methods Mol Biol **619:**145-156.

6. **Laue TM, Shah BD, Ridgeway TM, Pelletier SL.** 1992. Computer-aided interpretation of analytical sedimentation data for proteins, p. 90-125. *In* Harding SE, Rowe, A. J. & Horton, J.C., eds (ed.), Analytical Ultracentrifugation in Biochemistry and Polymer Sciences. Royal Society of Chemistry, Cambridge,UK.

7. **Schuck P, Rossmanith P.** 2000. Determination of the sedimentation coefficient distribution by least-squares boundary modeling. Biopolymers **54:**328-341.
