# Supplementary material for: Unraveling the Self-Assembly of the Pseudomonas aeruginosa XcpQ Secretin Periplasmic Domain Provides New Molecular Insights into Type II Secretion System Secreton Architecture and Dynamics
Source: mBio. 2017 Oct 17;8(5):e01185-17. doi: 10.1128/mBio.01185-17 (PMC5646246; doi:10.1128/mBio.01185-17)
Supplement: FIG S1 [file mbo005173532sf1.pdf]

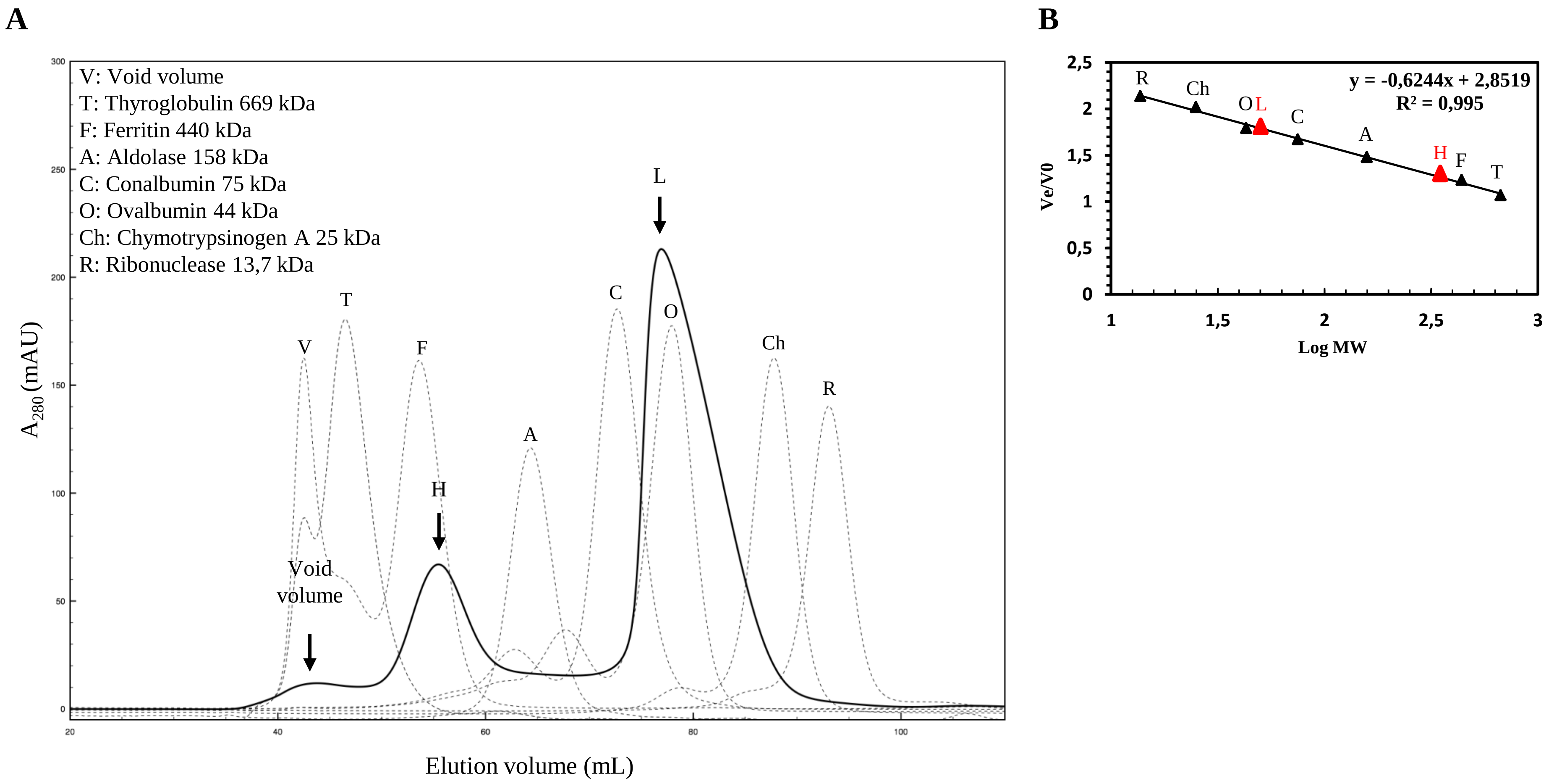

Figure S1. Analysis of the oligomeric state of $XcpQ_{N012}$ by SEC.

A. SEC curves using calibration standard proteins. The curve of the $XcpQ_{N012}$ was superimposed with calibration curves. The elution volume (from a HiLoad 16/600 Superdex 200 column) is plotted on the x axis, and the 280-nm absorbance is plotted on the y axis. The initials for each calibration standard proteins is mentioned as well as the corresponding molecular weight. **B.** HiLoad 16/600 Superdex 200 calibration. The log (MW) of standard proteins is plotted on the x axis and the calculated Ve/Vo is plotted on the y axis. Each dark triangle indicates the position of each protein used for the calibration. The red triangles indicate the position of H and L species.
