# Supplementary material for: Unraveling the Self-Assembly of the Pseudomonas aeruginosa XcpQ Secretin Periplasmic Domain Provides New Molecular Insights into Type II Secretion System Secreton Architecture and Dynamics
Source: mBio. 2017 Oct 17;8(5):e01185-17. doi: 10.1128/mBio.01185-17 (PMC5646246; doi:10.1128/mBio.01185-17)
Supplement: FIG S2 [file mbo005173532sf2.pdf]

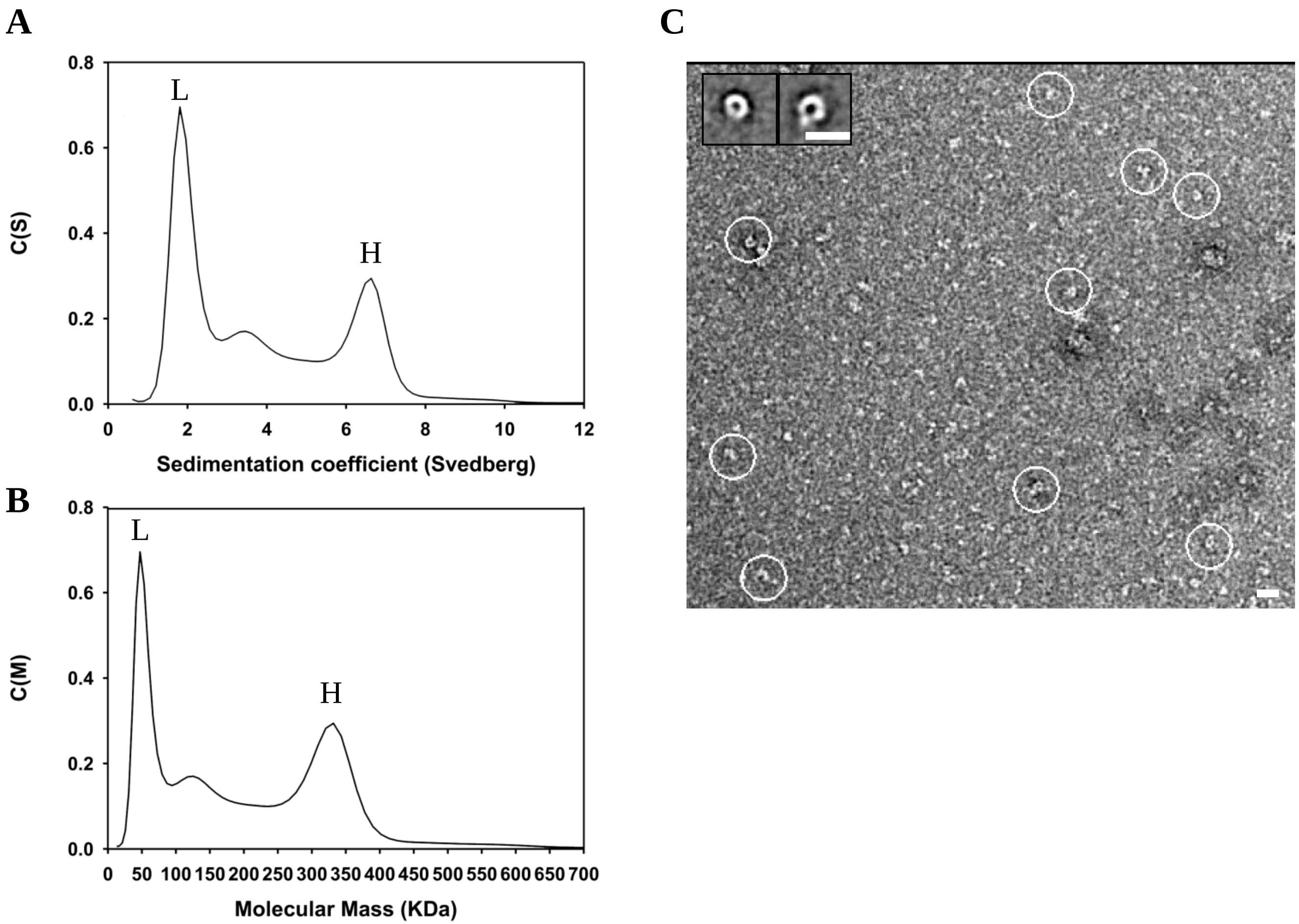

Figure S2. Analysis of the oligomeric state of XcpQ$_{N012}$ by AUC and EM.

A. Coefficient sedimentation distribution C(S) of 7 mg/mL of XcpQ$_{N012}$ obtained by sedimentation velocity experiments. **B.** The C(M) distribution function obtained from these similar experiment indicates the presence of species with molecular mass compatible with a dimer (L) and a dodecamer (H). **C.** Negative stain EM images of XcpQ$_{N012}$ oligomeric complex purified by SEC. The insets show two class average generated by EMAN2 from 166 picked particles. Scale bar (20 nm) is shown in white.
