# Supplementary material for: Unraveling the Self-Assembly of the Pseudomonas aeruginosa XcpQ Secretin Periplasmic Domain Provides New Molecular Insights into Type II Secretion System Secreton Architecture and Dynamics
Source: mBio. 2017 Oct 17;8(5):e01185-17. doi: 10.1128/mBio.01185-17 (PMC5646246; doi:10.1128/mBio.01185-17)
Supplement: FIG S3 [file mbo005173532sf3.pdf]

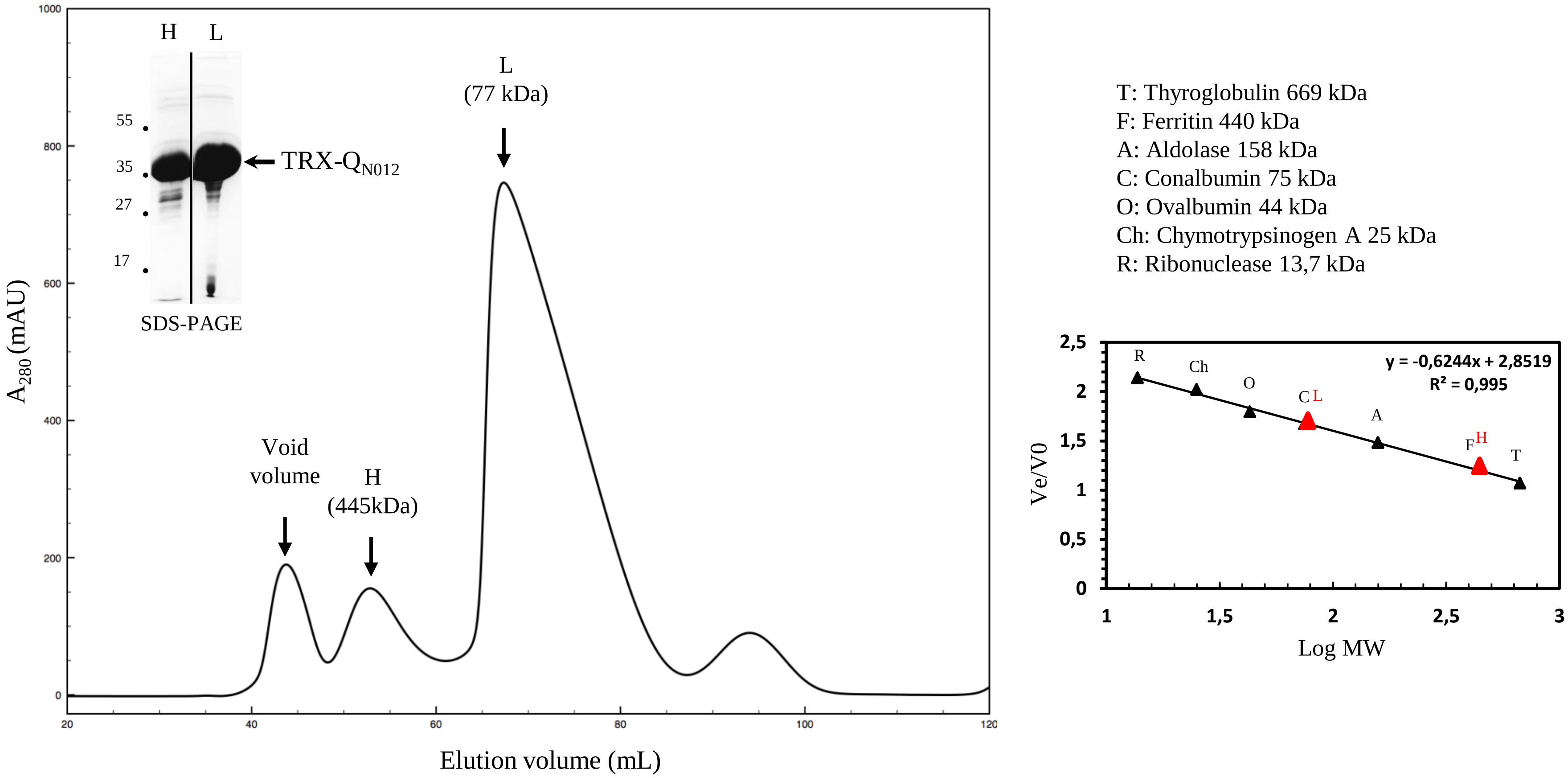

Figure S3. Trx-XcpQ$_{N012}$ assembles into a dodecameric complex.

SEC of the purified Trx-XcpQ$_{N012}$. The elution volume (from a Superdex 200 1660 column) is plotted on the x axis, and the 280-nm absorbance is plotted on the y axis. Left upper insert is the Coomassie-stained SDS-PAGE analysis of the H and L fractions. Right: estimation of the MW of the H and L species of Trx-Q$_{N012}$ using S200 1660 calibration. Dark triangles indicate the position of each protein used for the calibration. The red triangles indicate the position of H and L species. The estimated MW is indicated on the SEC profile.
