# Supplementary material for: Unraveling the Self-Assembly of the Pseudomonas aeruginosa XcpQ Secretin Periplasmic Domain Provides New Molecular Insights into Type II Secretion System Secreton Architecture and Dynamics
Source: mBio. 2017 Oct 17;8(5):e01185-17. doi: 10.1128/mBio.01185-17 (PMC5646246; doi:10.1128/mBio.01185-17)
Supplement: FIG S4 [file mbo005173532sf4.pdf]

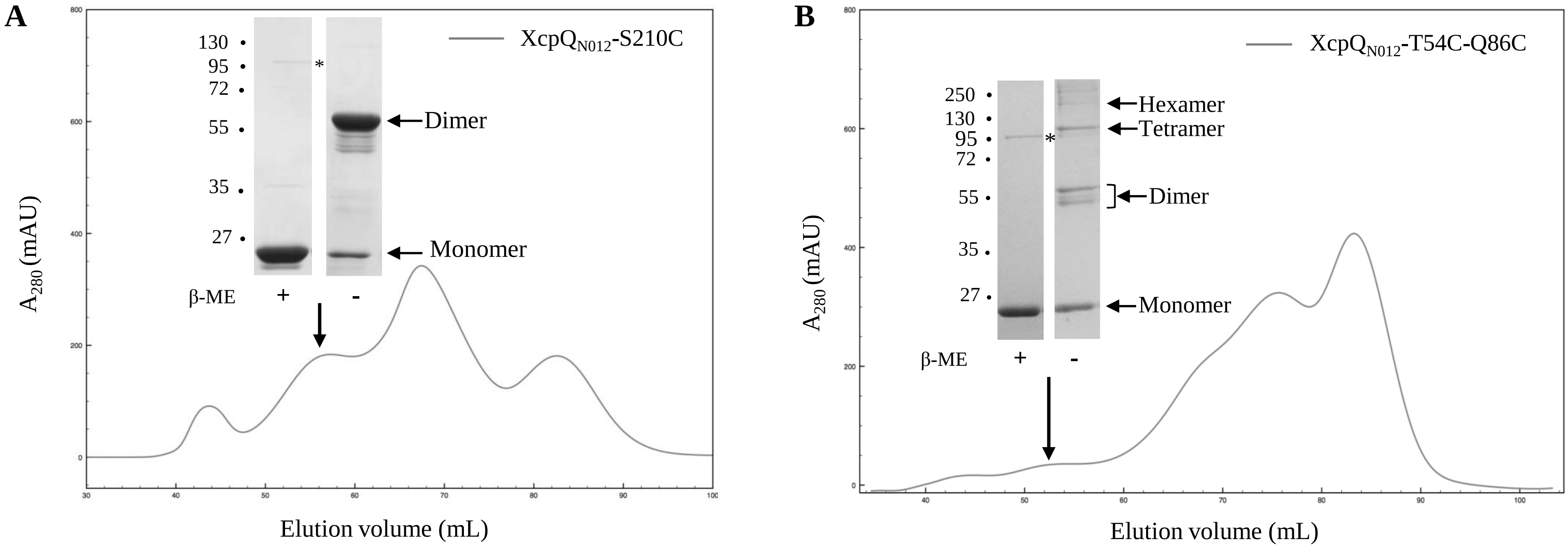

Figure S4. XcpQ$_{N012}$-S210C and XcpQ$_{N012}$-T54C-Q86C form oligomers in the dodecameric complex under oxidative conditions.
SEC of the purified XcpQ$_{N012}$-S210C (**A**) and XcpQ$_{N012}$-T54C-Q86C (**B**) variants. The fractions corresponding to the dodecameric complex of each variants indicated by an arrows were analyzed by Coomassie-stained SDS-PAGE under reducing and non-reducing conditions (+ and – β-mercaptoethanol (β-ME)). XcpQ$_{N012}$-S210C and XcpQ$_{N012}$-T54C-Q86C dimers and higher oligomers are indicated by arrows. The non-specific band recovered above 95 kDa in SEC experiments is indicated by a star (*). Molecular weight markers (in kDa) are indicated on the left.
