# Supplementary material for: Unraveling the Self-Assembly of the Pseudomonas aeruginosa XcpQ Secretin Periplasmic Domain Provides New Molecular Insights into Type II Secretion System Secreton Architecture and Dynamics
Source: mBio. 2017 Oct 17;8(5):e01185-17. doi: 10.1128/mBio.01185-17 (PMC5646246; doi:10.1128/mBio.01185-17)
Supplement: FIG S5 [file mbo005173532sf5.pdf]

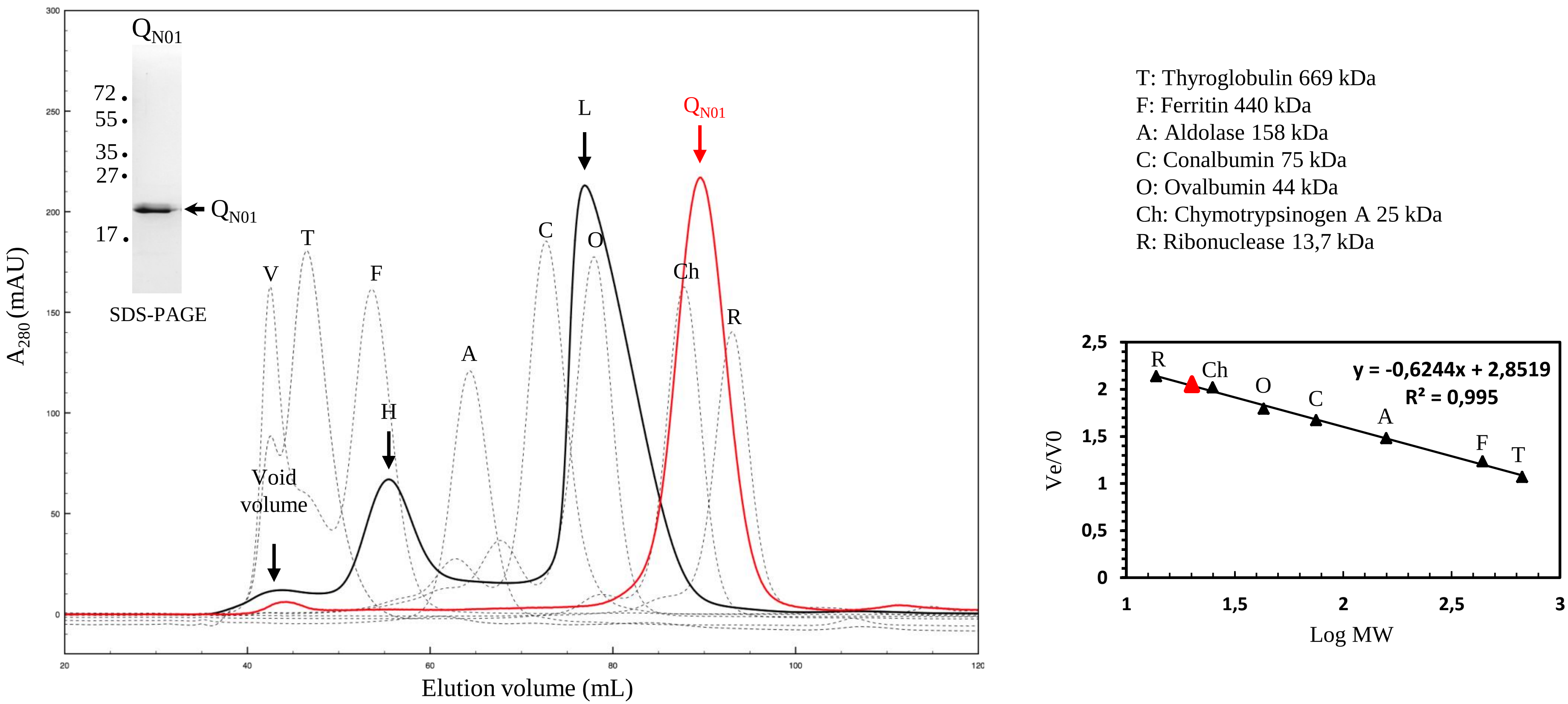

Figure S5: The N2 sub-domain is essential for XcpQ$_{N012}$ dodecamer assembly.

SEC profile of XcpQ$_{N012}$ (black line) was superimposed to XcpQ$_{N01}$ (red line). The SEC profile of the protein standards is also shown in dash lines. The calibration curve as well the position of the estimated MW of XcpQ$_{N01}$ (red triangle) is shown in the right panel.
