# Supplementary material for: Unraveling the Self-Assembly of the Pseudomonas aeruginosa XcpQ Secretin Periplasmic Domain Provides New Molecular Insights into Type II Secretion System Secreton Architecture and Dynamics
Source: mBio. 2017 Oct 17;8(5):e01185-17. doi: 10.1128/mBio.01185-17 (PMC5646246; doi:10.1128/mBio.01185-17)
Supplement: FIG S6 [file mbo005173532sf6.pdf]

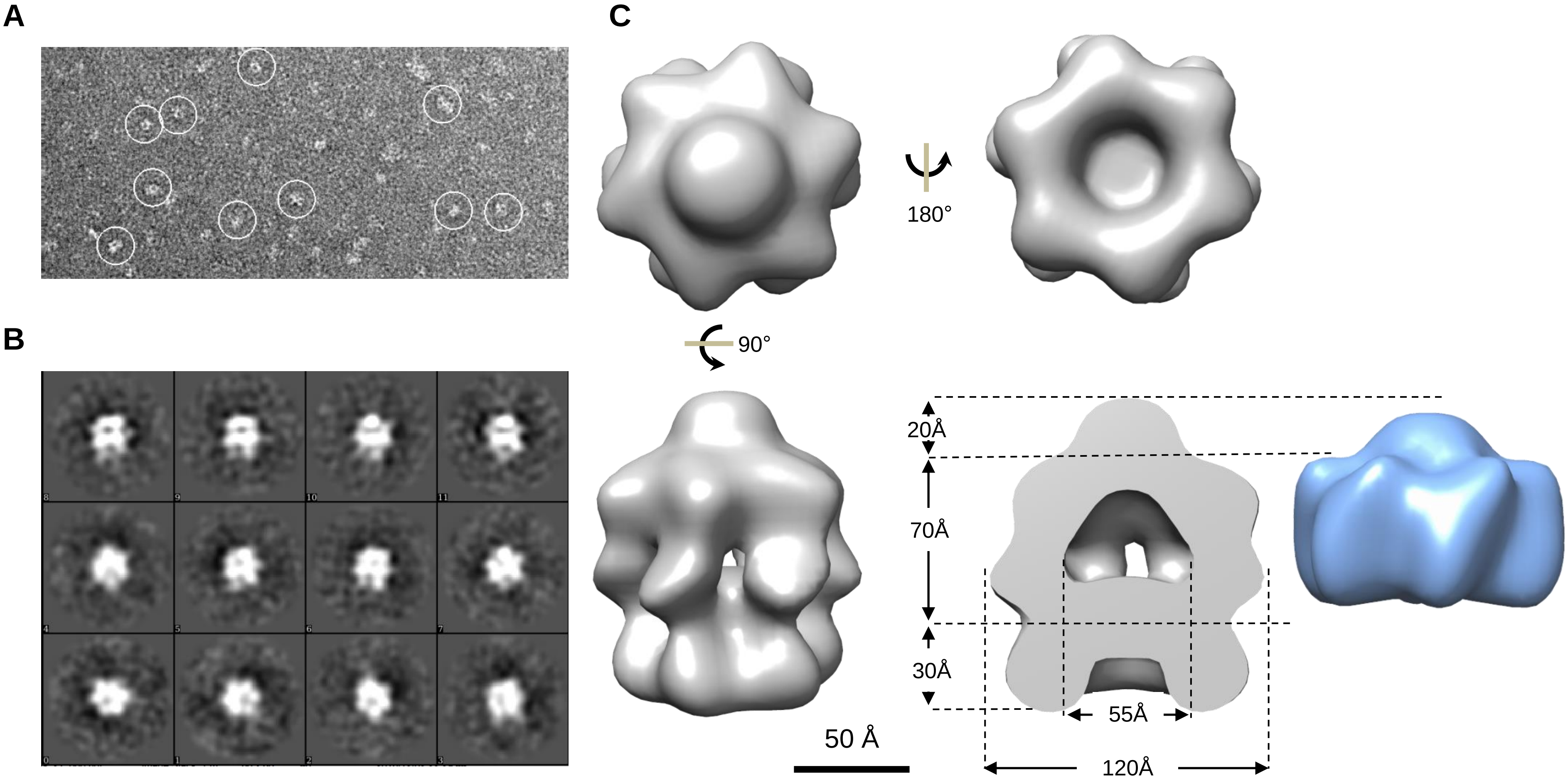

Figure S6: Low-resolution EM model of $XcpQ_{N012}$-S210C.

a. Representative micrograph of the data set used for image processing. White circles indicate isolated Trx-$XcpQ_{N012}$-S210C dodecamers. **b.** Gallery of representative class averages generated by EMAN2 after 2D classification. **c.** Top, side and bottom views of the three-dimensional reconstruction model of the $XcpQ_{N012}$-S210C dodecamer obtained by electron microscopy (accession number: EMD-3649). The three-dimensional reconstitution of $XcpQ_{N012}$ colored in violet in also shown. Scale bar (5 nm) is shown in black.
