# Supplementary material for: Unraveling the Self-Assembly of the Pseudomonas aeruginosa XcpQ Secretin Periplasmic Domain Provides New Molecular Insights into Type II Secretion System Secreton Architecture and Dynamics
Source: mBio. 2017 Oct 17;8(5):e01185-17. doi: 10.1128/mBio.01185-17 (PMC5646246; doi:10.1128/mBio.01185-17)
Supplement: FIG S7 [file mbo005173532sf7.pdf]

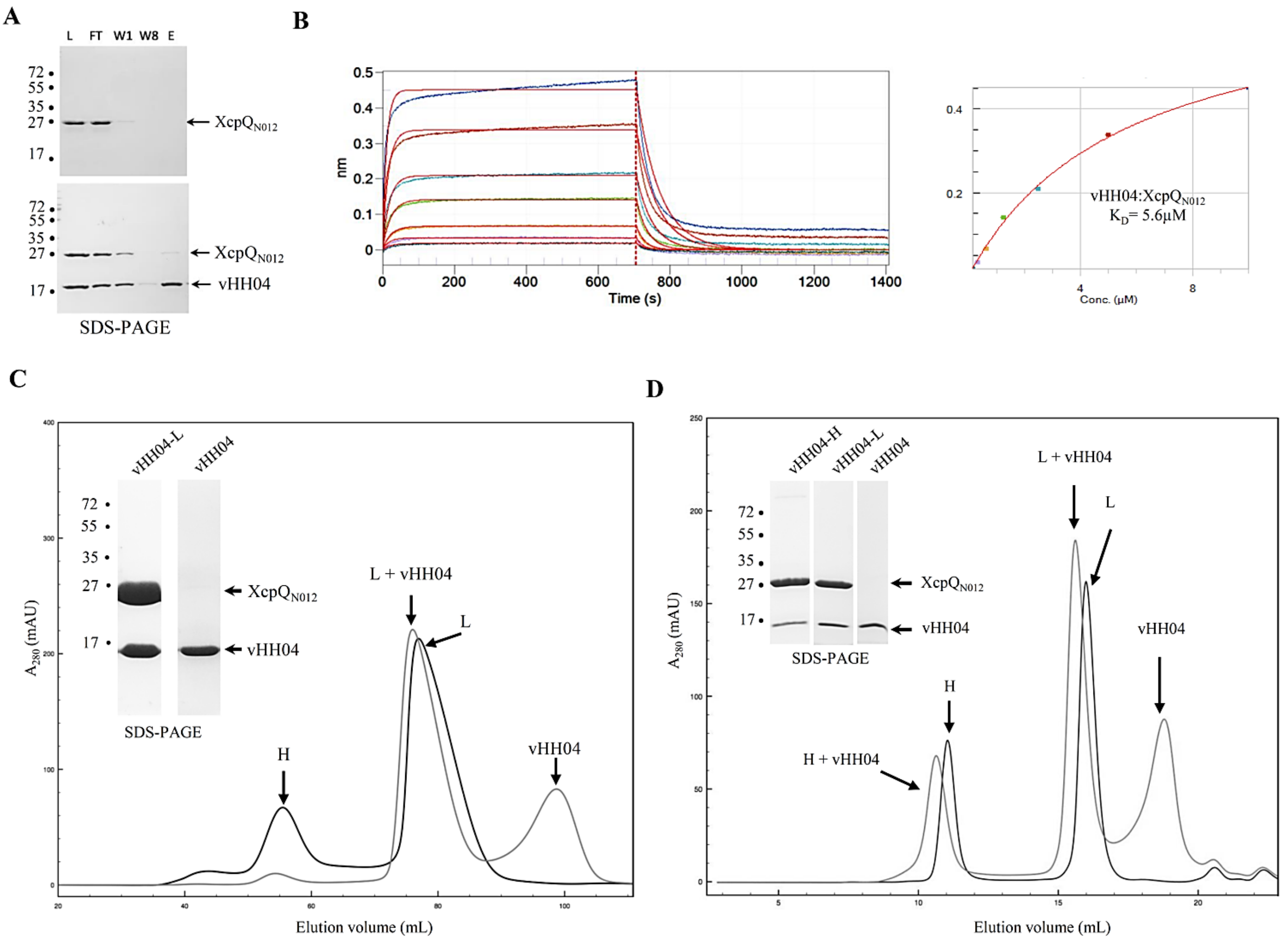

Figure S7. Identification and binding properties of vHH04 nanobodies to $XcpQ_{N012}$. A. Co-purification experiments of purified $XcpQ_{N012}$ with his-tagged vHH04. L: loading material, FT: Flow through, W1: Wash1, W8: Wash 8, E: Elution. The presence of untagged proteins in the elution fractions indicates its direct interaction with the vHH04. **B.** Characterization of vHH04/$XcpQ_{N012}$ binding using Bio-Layer Interferometry (BLi). Colored curves represent the association and the dissociation of increasing concentrations of soluble vHH04 (from 0,156 to 10 µM) on $XcpQ_{N012}$ fixed on the streptavidin sensors using Biotin tag. The red curves represent the statistical fitting of the experimental curves. vHH04 binds $XcpQ_{N012}$ with µM range. **C**. SEC profile of purified vhh04 mixed with the isolated $XcpQ_{N012}$ dimers and superimposed with the SEC of purified $XcpQ_{N012}$. Peaks corresponding to the isolated complexes were analyzed by 15% SDS-PAGE/Coomassie (left insets). The L and H letters indicates the $XcpQ_{N012}$ dimeric and dodecameric complexes. **D**. SEC profile of purified vHH04 independently mixed with the oligomeric $XcpQ_{N012}$ (grey line) and superimposed with the SEC of purified $XcpQ_{N012}$ alone (black line). Peaks corresponding to the isolated complexes were analyzed by 15% SDS-PAGE/Coomassie (left inset). The L and H letters indicates the $XcpQ_{N012}$ dimeric and dodecameric complexes. Molecular weight markers (in kDa) are indicated on the left.
