# Supplementary material for: Unraveling the Self-Assembly of the Pseudomonas aeruginosa XcpQ Secretin Periplasmic Domain Provides New Molecular Insights into Type II Secretion System Secreton Architecture and Dynamics
Source: mBio. 2017 Oct 17;8(5):e01185-17. doi: 10.1128/mBio.01185-17 (PMC5646246; doi:10.1128/mBio.01185-17)
Supplement: TABLE S1 [file mbo005173532st1.docx]

**Table S1A.** Plasmids used in this study

| Name | Description | Reference |
| --- | --- | --- |
| pETG-20A | Amp^R^ pBR322-derived ColE1, T7/lac, expression vector | A. Gerloof (EMBL) |
| pETG-20A-XcpQ_N012_ | *xcpQ_N012_* gene fragment cloned into pETG-20A | (1) |
| pETG-20A-XcpQ_N01_ | *xcpQ_N01_* gene fragment cloned into pETG-20A | (1) |
| pETG-20A-XcpPp | *xcpP_p_* gene fragment cloned into pETG-20A | (1) |
| pHEN6 | Phage display vector | (2) |
| pHEN6-vHH04 | *vHH04* gene fragment cloned into pHEN6 | This study |
| pMMB67HE | Amp^R^ (Cb^R^) p*tac*  broad-host-range vector | (3) |
| pMMB67HE-vHH04 | *vHH04* gene fragment cloned into pMMB67HE | This study |
| pET-DUET-1 | pBR322-derived ColE1, lacI, Amp^R^. | Invitrogen |
| pCbpD | cbpD Amp^R^, pT7.5 containing the C-terminal *cbpD-His10* DNA sequence | (4) |
| pET-DUET-CbpD | *cbpD-His10* DNA sequence subcloned from pCbpD at EcoRI site in pET-DUET-1 vector | This study |
| pRK2013 | Km^R^, ColE1, Tra+ Mob+ (RK2) | (5) |
| pJN105 | Gm^R^, *araC*-pBAD, broad-host-range vector | (6) |
| pJN-XcpQ | *xcpQ* gene fragment cloned into pJN105 | This study |
| pJN-XcpQ-S210C | *xcpQ-S210C* gene fragment cloned into pJN105 | This study |
| pJN-XcpQ-T54C | *xcpQ-T54C* gene fragment cloned into pJN105 | This study |
| pJN-XcpQ-Q86C | *xcpQ-Q86C* gene fragment cloned into pJN105 | This study |
| pJN-XcpQ-T54C-Q86C | *xcpQ-T54C-Q86C* gene fragment cloned into pJN105 | This study |

**Table S1B.** Oligonucleotides used in this study

| **Primer name** | **Primer sequence** |
| --- | --- |
| *xcpQ*-F  *xcpQ*-R | 5’-gaattcctgcagcccgggatcgcagaacccgccga-3’  5’-agaactagtggatcccccgggttacgtagaatcgagaccgaggagagggttagggataggcttaccttccgtcatcagttcgcg-3’ |
| *xcpQ*-T54C-F  *xcpQ*-T54C-R | 5’-caggaggcgcactggtgcatcaacctcaaggat-3’  5’- atccttgaggttgatgcaccagtgcgcctcctg-3’ |
| *xcpQ*-Q86C-F  *xcpQ*-Q86C-R | 5’-ccgcgggtcaagggctgcgtcagcgtggtctcc-3’  5’-ggagaccacgctgacgcagcccttgacccgcgg-3’ |
| *xcpQ*-S210C-F  *xcpQ-*S210C-R | 5’-gcagccacgactactgcgtgatcaacctg-3’  5’-caggttgatcacgcagtagtcgtggctgc-3’ |

**References**

1. **Douzi B, Ball G, Cambillau C, Tegoni M, Voulhoux R.** 2011. Deciphering the Xcp *Pseudomonas aeruginosa* type II secretion machinery through multiple interactions with substrates. J Biol Chem **286:**40792-40801.

2. **Desmyter A, Farenc C, Mahony J, Spinelli S, Bebeacua C, Blangy S, Veesler D, van Sinderen D, Cambillau C.** 2013. Viral infection modulation and neutralization by camelid nanobodies. Proc Natl Acad Sci U S A **110:**E1371-1379.

3. **Furste JP, Pansegrau W, Frank R, Blocker H, Scholz P, Bagdasarian M, Lanka E.** 1986. Molecular cloning of the plasmid RP4 primase region in a multi-host-range tacP expression vector. Gene **48:**119-131.

4. **Cadoret F, Ball G, Douzi B, Voulhoux R.** 2014. Txc, a new type II secretion system of *Pseudomonas aeruginosa* strain PA7, is regulated by the TtsS/TtsR two-component system and directs specific secretion of the CbpE chitin-binding protein. J Bacteriol **196:**2376-2386.

5. **Figurski DH, Helinski DR.** 1979. Replication of an origin-containing derivative of plasmid RK2 dependent on a plasmid function provided in trans. Proc Natl Acad Sci U S A **76:**1648-1652.

6. **Newman JR, Fuqua C.** 1999. Broad-host-range expression vectors that carry the L-arabinose-inducible *Escherichia coli* araBAD promoter and the araC regulator. Gene **227:**197-203.
